# A biological ocean data reformatting effort

**DOI:** 10.1038/s41597-024-03038-0

**Published:** 2024-02-16

**Authors:** Kimberlee Baldry, Robert Johnson, Peter G. Strutton, Philip W. Boyd

**Affiliations:** 1 Institute for Marine and Antarctic Studies, University of Tasmania, Hobart, Australia; 2 Bureau National Operations Centre, Bureau of Meteorology, Hobart, Australia; 3 Australian Centre for Excellence in Antarctic Science, University of Tasmania, Hobart, Australia; 4 Australian Research Council Centre of Excellence for Climate Extremes, University of New South Wales, Sydney, Australia

**Keywords:** Carbon cycle, Marine biology

## Abstract

Biological ocean data collected from ships find reuse in aggregations of historical data. These data are heavily relied upon to document long term change, validate satellite algorithms for ocean biology and are useful in assessing the performance of autonomous platforms and biogeochemical models. Existing aggregate products have largely been restricted to the surface ocean, omit physical data or have limited biological data. We present the first version of a BIOlogical ocean data reforMATting Effort (BIO-MATE) to begin to fill a gap in subsurface bio-physical data aggregates in a reproducible way. BIO-MATE uses open-source R software that reformats openly sourced published datasets from oceanographic voyages. These reformatted biological and physical data from underway sensors, profiling sensors, pigments analysis and particulate organic carbon analysis are stored in an interoperable BIO-MATE data product for easy access and use. Specific QA/QC protocols can now be easily applied to the BIO-MATE data product to support a variety of surface and subsurface applications.

## Background & Summary

Marine phytoplankton blooms support ocean food-webs and influence global climate through the biological carbon pump^1–3^. Ocean physics and other environmental drivers control the timing, magnitude and extent of phytoplankton blooms through complex bio-physical relationships^4–7^. To study these relationships, integrated data structures that link biological and physical ocean data are needed. Ship-based data are the gold standard for accurate biological oceanographic measurements^8^. These data are often published separately to physical ocean data, stored across different repositories and in multiple formats. This makes it difficult and time-consuming to aggregate and link biological and physical data. The described data product attempts to make this task easier.

**Fig. 1:**
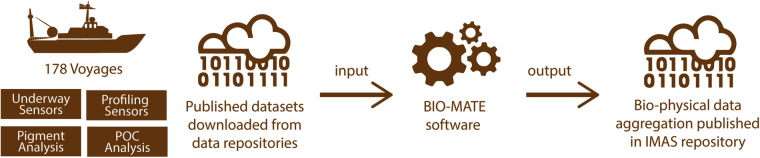
The BIO-MATE concept for creating a consistent data compilation from existing ship-based oceanographic data.

The biological ocean data reformatting effort (BIO-MATE) works to link existing, open-access biological and physical datasets across oceanographic voyages and promote their re-use (Fig. 1). This has been done by developing a BIO-MATE R software package that not only reformats published datasets, but also cross-references between biological and physical data and allows access to citation information (https://github.com/KimBaldry/BIOMATE-Rpackage). The resulting BIO-MATE data product allows users to easily access, manipulate and cite published ship-based datasets of different dimensions for multiple applications.

The BIO-MATE data product can be accessed via the IMAS Data Portal^9^ and the Australian Ocean Data Network (https://portal.aodn.org.au/). The aggregation includes four data streams: (1) data collected from shipboard underway sensors, (2) profiling sensors mounted on sampling rosettes, (3) lab analysis for phytoplankton pigments and (4) lab analysis for particulate organic carbon (POC). These data streams are cross-referenced by unique expedition codes (EXPOCODE) and profiling station identifications (CTD_ID). An additional data stream contains supporting information for the data product including a list of oceanographic voyages, investigator contact information and data citations for reformatted datasets. We have also included an aggregated data table for biological data. Users are requested to refer to supporting data and cite all data products accessed through BIO-MATE, as well as the BIO-MATE data product itself. We consulted the distribution licenses of all data sources to ensure that with this condition data are re-used lawfully.

The data product has been used to understand how the response of in-situ fluorometers changes in the Southern Ocean, to assess non-photochemical quenching corrections and to investigate the role of ocean physics in mediating subsurface chlorophyll features^10^. These examples highlight the malleability of this data product to improve our understanding of biological oceanography. Other potential applications include validating satellite observations^11,12^, developing new ways to validate in-situ bio-optical observations collected by autonomous profiling platforms in the presence of dynamic fronts^8,13,14^, training ocean state estimations^15^, informing bio-physical models and using multi-variate analyses to understand bio-physical relationships.

We recognise the massive effort in producing the thousands of data records in this data product. This includes the investigators and data officers who have spent countless hours in ship time, project organisation, grant writing, laboratory analysis, data processing and report writing. Oceanographic data are often collected with regional studies in mind, but their value increases with publication and re-use. We encourage all investigators to publish their data for re-use through data products like BIO-MATE.

## Methods

### Published datasets in BIO-MATE

**Fig. 2:**
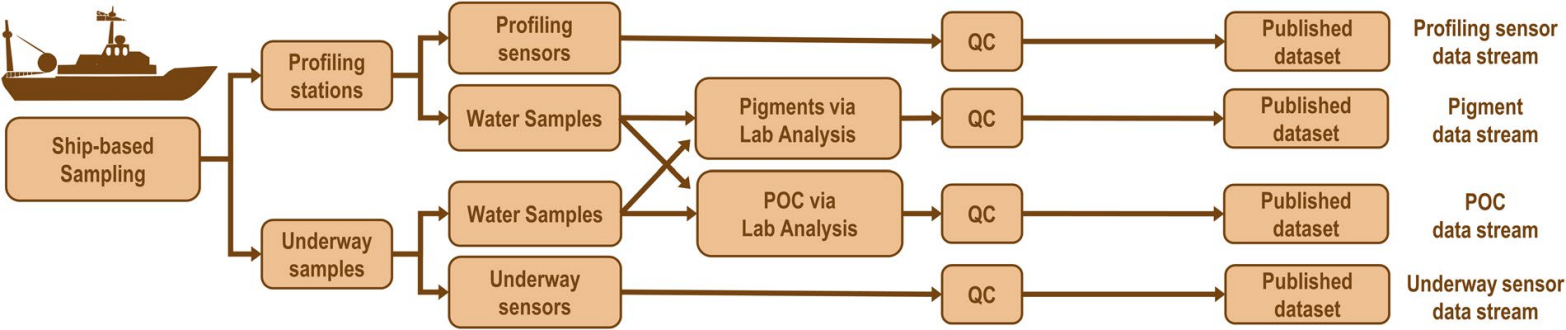
Typical data collection and treatment process for biological oceanographic data within the BIO-MATE data compilation.

The BIO-MATE aggregate data product^9^ brings together ship-based data that have been collected by a Principal Investigator (PI), published to a publicly accessible database and re-cited in this data descriptor^16–506^ (Fig. 2). The first version of BIO-MATE includes published datasets associated with four types of measurements:

1. sensors in the vessels underway seawater in-take (underway sensor data stream),
2. profiling sensors mounted to sampling rosettes (profiling sensor data stream),
3. pigments measured in the laboratory (pigment data stream), and
4. POC measured in the laboratory (POC data stream).

Data records from the pigment data stream were first identified in data repositories that host biological data. Pigment data records were identified using the search term “chlorophyll” and a latitude bound of 30 to 90 S from PANGAEA^506^ (https://www.pangaea.de/), SeaBASS^500,501^ (https://seabass.gsfc.nasa.gov/), the Australian Ocean Data Network (AODN, https://portal.aodn.org.au/), GLODAP^466,467^ (https://www.glodap.info/), the Palmer Long Term Ecological Record (PAL-LTER, https://pal.lternet.edu/data), the Biological and Chemical Oceanography Data Management Office (BCO-DMO, https://www.bco-dmo.org/data), the CSIRO Marlin Data Trawler (Marlin, https://www.cmar.csiro.au/data/trawler/) and the Australian Antarctic Data Center (AADC, https://data.aad.gov.au/). Data records from the profiling sensors and underway sensors data streams were then identified in these repositories and in the CCHDO (https://cchdo.ucsd.edu/) and MGDS^493^ (https://www.marine-geo.org/). Although we largely constrained the first version of BIO-MATE aggregate data product to the Southern Ocean, further versions can be expanded to the global ocean.

From available pigment data records, 178 relevant voyages were identified using unique 12-digit expedition codes (EXPOCODES) assigned as follows; National Oceanographic Data Centre (NODC) platform codes followed by voyage 8 digit start dates (YYYYMMDD). NODC platform and country codes are recorded on Git Hub (https://github.com/KimBaldry/BIO-MATE/product_data/supporting_information/codes) and within the BIO-MATE software (https://github.com/KimBaldry/BIOMATE-Rpackage/inst/codes). If the vessel name or voyage start/end dates were absent, this information was found using Google to discover voyage records. This voyage information was used to do a final Google keywords search (i.e. ship name, synonyms for voyages, year, “underway”, “CTD”, “chlorophyll”,”POC”, “cruise report” and “data”) to determine any absent records and to discover accompanying cruise reports.

### Semi-automated BIO-MATE workflow for reformatting datasets

**Fig. 3:**
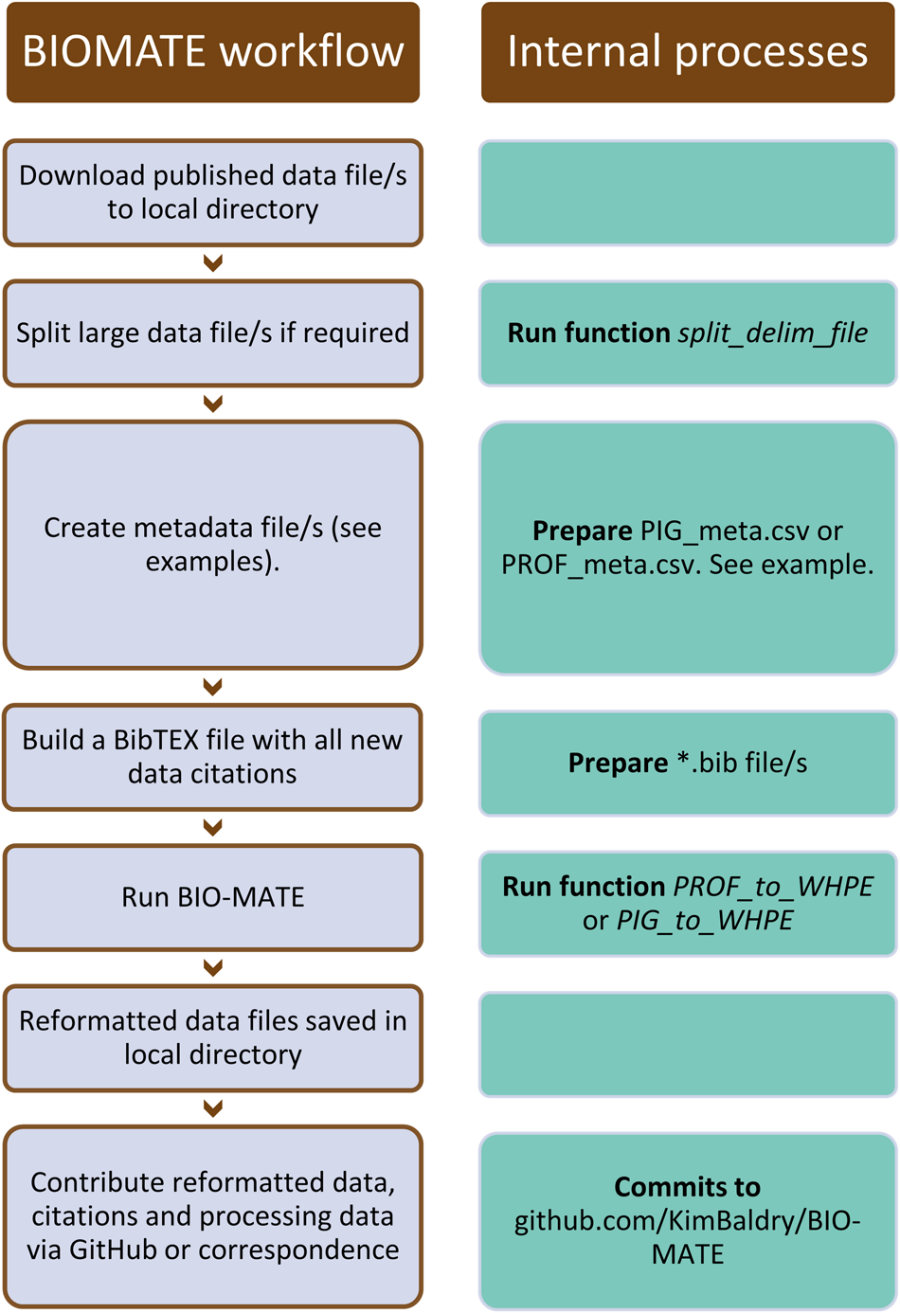
A schematic demonstrating the BIO-MATE workflow.

A semi-automated workflow and the BIO-MATE R software (https://github.com/KimBaldry/BIOMATE-Rpackage) were used to reformat published datasets, and produce the BIO-MATE data product (Fig. 3). Downloaded data files were split by EXPOCODES if they recorded data within a larger dataset (e.g PAL-LTER data records). Files for the profiling sensor data stream were further split into individual profiles. Processing metadata were manually entered into a table to inform the BIO-MATE R software and a bulk run of the software was performed to reformat data files. The workflow is described in more detail in the following subsections (Fig. 2).

### Download of published datasets

Published datasets were manually downloaded from open source repositories and stored locally in accordance with data policies. Some manual reformatting of a small portion of downloaded data had to be performed on old datasets, prior to the application of reformatting scripts, due to formatting irregularities. Downloaded data files, and their amendments used to create the BIO-MATE data product, are not published in BIO-MATE, but are available upon request to the corresponding author.

### Splitting large datasets with BIO-MATE software

The BIO-MATE R software requires each file to only contain observations from a single voyage. Further, the profiling sensor data stream requires each file to only contain observations from a single profiling cast, held in a discrete directory for each voyage.

The *split_delim_file* function splits files using identified variables containing EXPOCODE synonyms and/or profiling station information. This function can be used to split a single, large data file into smaller files as required. For this version of the data product, a number of files had to be split to be ingested into the BIO-MATE core functions. A record of these can be found in Git Hub in the project notebook (https://github.com/KimBaldry/BIO-MATE/blob/main/BIO-MATE.Rmd).

### Processing metadata

Information on file formats, dataset information, citation information, location data variables and ocean data variables are needed to reformat published datasets with BIO-MATE software. This information is called processing metadata herein and was manually entered and stored as comma delimited text files. The processing metadata required to run BIO-MATE software is described in the supplement Processing Metadata Table and differs for each data stream. All processing metadata used to construct the BIO-MATE aggregated data product is stored in Git Hub (https://github.com/KimBaldry/BIO-MATE/tree/main/product_data/processing_metadata).

### Dataset citation with BibTEX files

Information is included in the BIO-MATE data product, for citing published datasets, laboratory analysis methodologies (for the pigment and POC data streams) and the data repositories through which published data records were accessed. Each citation was recorded as a BibTeX entry, compatible with EndNote, R and LaTeX. Each BibTeX entry has a tag that is referenced in the processing metadata. This tag is used to link citations to their corresponding data records when datasets are ingested in the BIO-MATE software. Citation information is then printed in the header information in reformatted files. Where possible BibTeX entries were sourced from data repositories. If BibTeX entries were not found, they were created manually.

All BibTeX entries are stored on Git Hub (https://github.com/KimBaldry/BIO-MATE/product_data/supporting_information/citations) and in the BIO-MATE software (https://github.com/KimBaldry/BIOMATE-Rpackage/inst/citations). A look-up table is included in the BIO-MATE software to help users find relevant BibTeX entries needed to cite datasets appropriately (https://github.com/KimBaldry/BIOMATE-Rpackage/tree/main/data). A function *export_ref* supports the export of a smaller BibTeX file based on user selections of EXPOCODES and data streams that they have accessed through the product. This allows references to be easily appended to a bibliography as required.

### Reformatting and linking data streams with BIO-MATE R software

The BIO-MATE R software was run to reformat data files to the WHP (CCHDO)-Exchange format (https://exchange-format.readthedocs.io/en/latest/index.html), using the original or split data files, processing metadata and citation information as input. The software arranges reformatted WHPE files into four data streams in local directories that include separate WHPE files, for each EXPOCODE, and for underway sensors, profiling sensor casts, pigment measurements, and POC measurements.

Each data stream has its own reformatting function within the BIO-MATE R software (*UWY_to_WHPE*, *PROF_to_WHPE*, *PIG_to_WHPE*, *POC_to_WHPE*). The software requires physical (underway sensor and profiling sensor) data streams to be reformatted before biological (pigment and POC) data streams, to accommodate a biological-physical matching algorithm within the PIG_to_WHPE and POC_to_WHPE functions. The algorithm links biological data in the pigment and POC data streams to the physical data in the profiling sensor and underway sensor data streams. Biological data records are given a profiling sensor identification tag (CTD_ID) if matched to physical data in BIO-MATE.

To match biological data to physical data, the algorithm first uses EXPOCODES to find relevant physical data in profiling sensor data streams. It then matches biological and physical data records by comparing station number (STNBR) and cast number (CASTNO) records. If matches are detected using STNBR and CASTNO, the validity of these matches is checked by comparing time and position information, but if position and time were not recorded in biological datasets (4157 pigment and 1948 POC records) it is assumed that the STNNBR and CASTNO records are correct if they match between data streams (e.g. in the JGOFS records). If position or time was recorded, a check on identified matches is performed to see if both the biological and physical data record data either within 24 hours of each other or within 8 km^507^. This quick check catches cases where STNNBR and CASTNO are used in similar ways within physical and biological sampling, but exact matches do not correspond to the same sampling event.

If matches couldn’t be identified using STNBR and CASTNO between datasets a more rigorous search was performed using a database of time and position information from all profiling sensor data relating to the EXPOCODE. Matches were then found for biological data, if it contains position information, by finding the closest profiling sensor record within 1km in the database. If time information exits, matches are identified as the closest profiling sensor record within 6 hours, otherwise only matching date information is required. These position and time constraints are tighter than if STNNBR and CASTNBR records were matched. Using this process all biological sampling events had matching physical sampling events. Matching has only been implemented with physical profiling sensor data and not to physical underway data. Underway surface data do not require station IDs and are more simply found with EXPOCODE and position and/or time.

### Quality assurance

Limited quality assurance has been performed on the BIO-MATE data product and is variable across published datasets. As a supplement we include some insights into the quality of pigment data and chlorophyll fluorescence profiles which has been obtained through visual inspection (Supplementary QA/QC). The initial integrity of these data records lies with the Principal Investigators of the published data record. As a result, reformatted data have varying levels of quality control and post-processing. We have included cruise report citations in our product to aid in further data quality assurance efforts.

This allows a range of users to benefit from the BIO-MATE aggregate product and ensures data quality remains at the standard it was published. The quality assurance required of physical and biological ocean data varies according to application and is up to the user to confirm the data is suitable for their application. Future versions of BIO-MATE could implement quality assurance metrics under community consensus. The data can now be easily ingested by other data synthesis efforts, like GLODAP^466,467^ and the World Ocean Database (WOD), which implement established QA/QC protocols.

## Data Records

**Fig. 4:**
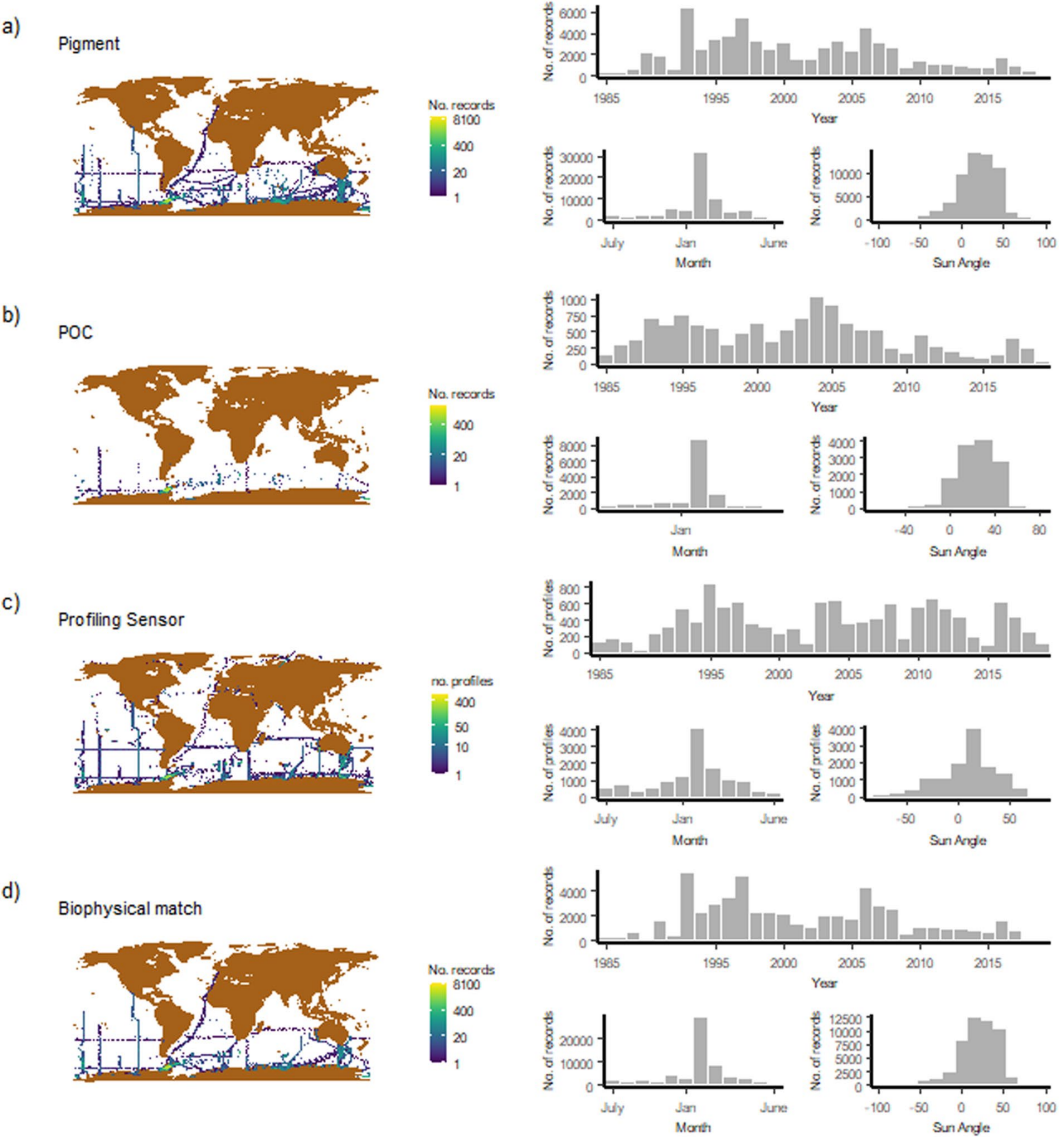
The spatiotemporal distribution of different data streams and bio-physical matches in the BIO-MATE data compilation.

The BIO-MATE data product^9^ is stored on the IMAS data portal (https://data.imas.utas.edu.au) and available on the AODN (https://portal.aodn.org.au/), formatted as four data streams linked through unique EXPOCODES. Supporting data contains a metadata table and BibTeX citation files. The spatial extent of the data records is confined largely to the Southern Ocean and was collected from 1985–2018 (Fig. 4). A summary of the data records in the BIO-MATE aggregate data product is presented in Tables 1–2.

**Table 1:** Summary of the pigment data records contained in the first version of BIO-MATE.

Statistic	All pigment records	Subsurface profile records (>4 depth samples above 75 m)	Surface records (<10 m)
Number of voyages	174	94	146
Number of records	65,834	38,458	18,025
Unique samples (no replicates)	62,020	36,095	15,916
Unique samples in lat and lon (not depth)	14,981	4,726	10,432
Bio-physical matches	50,048	38,458	12,003
HPLC and fluorometry matches	3,008	1,429	851
Number of HPLC records	27,299	13,667	7,513
Number of Fluorometry records	38,717	24,993	10,520
Matches with CTDFLUOR records	25,787	20,073	5,862
Matches with CTDBBP700 records	2,829	2,460	355
Matches with CTDBEAMCP records	6,980	5,777	917

**Table 2:** Summary of the profiling sensor data contained within the first version of BIO-MATE.

Number of voyages	127.00
Number of profiles	11,818.00
Profiles with pressure records (%)	100.00
Profiles with salinity records (%)	99.87
Profiles with temperature records (%)	99.90
Profiles with oxygen records (%)	42.44

### Underway sensor data stream

The underway sensor data stream contains a comma delimited WHP-Exchange file for each voyage ([EXPOCODE]_UWY.csv). The format of this file consists of headers to store metadata, followed by a data table that reports records collected by underway sensors mounted on the vessel (Data Records Table 1).

### Profiling sensor data stream

The profiling sensor data stream contains a comma delimited WHP-Exchange file for each unique profiling cast conducted on each voyage ([EXPOCODE]*[station number]*[cast number]_ctd1.csv). The file is formatted to store metadata as headers which is followed by the data table that reports records from profiling sensors mounted on a sampling rosette (Data Records Table 2).

### Pigment data stream

The pigment data stream contains a comma delimited WHP-Exchange file for each voyage (named [EXPOCODE]*PIG*[SOURCED_FROM]_[METHOD].csv). The format of this file consists of headers to store supporting information, followed by a data table that records measurements from the laboratory analysis of seawater samples for pigments performed by principal investigators (Data Records Table 3). The laboratory analyses considered are fluorometric determination and high-performance liquid chromatography (HPLC).

### Particulate organic carbon data stream

The POC data stream contains a comma delimited WHP-Exchange file for each voyage (named [EXPOCODE]*POC*[SOURCED_FROM]_[METHOD].csv). The format of this file consists of headers to store supporting information, followed by a data table that records measurements from the lab analysis of seawater samples for particulate organic carbon performed by principal investigators (Data Records Table 4).

### Supporting data

Supporting data are included in the BIO-MATE aggregate data product to support the correct citation of data and guide user access to data. This data includes (1) A BibTeX file, that contains information to reference all BIO-MATE data records (2) An index table indicating data availability and citation tags against data records listed by EXPOCODE, data stream, method and source, (3) A records table for all data repositories from which BIO-MATE data was sourced and (4) A records table for all pigment and POC analysis methods used in BIO-MATE data.

## Technical Validation

We validated the quality of the BIO-MATE data compilation, by displaying a number of key data distributions and trends. This validation does not confirm the quality of individual data points, in which the authors have placed no additional quality assurance to the published datasets.

**Fig. 5:**
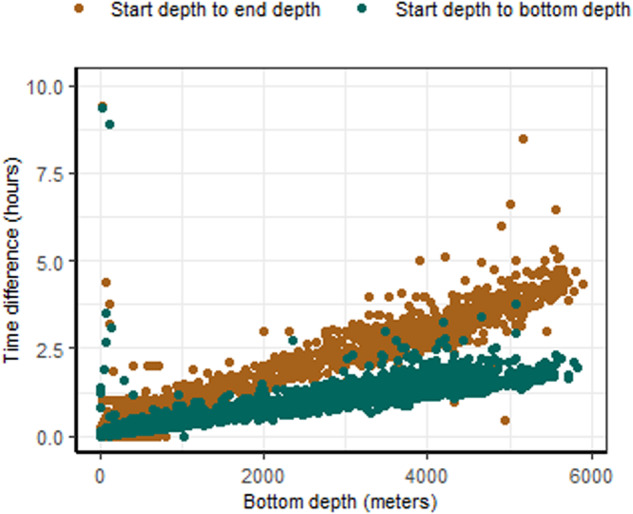
The time difference between the bottom depth (i.e. deepest position on the cast) and end depth (i.e last sampling position) of a profiling sensor cast versus the bottom depth of the cast. Outliers with a bottom depth close to 0, likely represent shallow testing or calibration casts.

The location data associated with the published datasets has been interpreted correctly by the software. This is evident from the success of the biophysical matching algorithm, along with the spatial distribution of the data and recorded sampling depths (Fig. 4). The data are predominantly collected in the month of January between 1991–2010. This is consistent with the fact that ship-based sampling in the Southern Ocean is conducted during Austral summer and displays a lag time in publishing most recent datasets to data repositories. All data are in the ocean, not on land, confirming the absence of spurious location data, and most samples are located in the Southern Ocean which is consistent with our search constraints. Finally information on sampling time of ship-based biological data is as expected, and CTD sampling times (start, bottom and end) are sequential and follow a trend with sampling depth (Fig. 5).

**Fig. 6:**
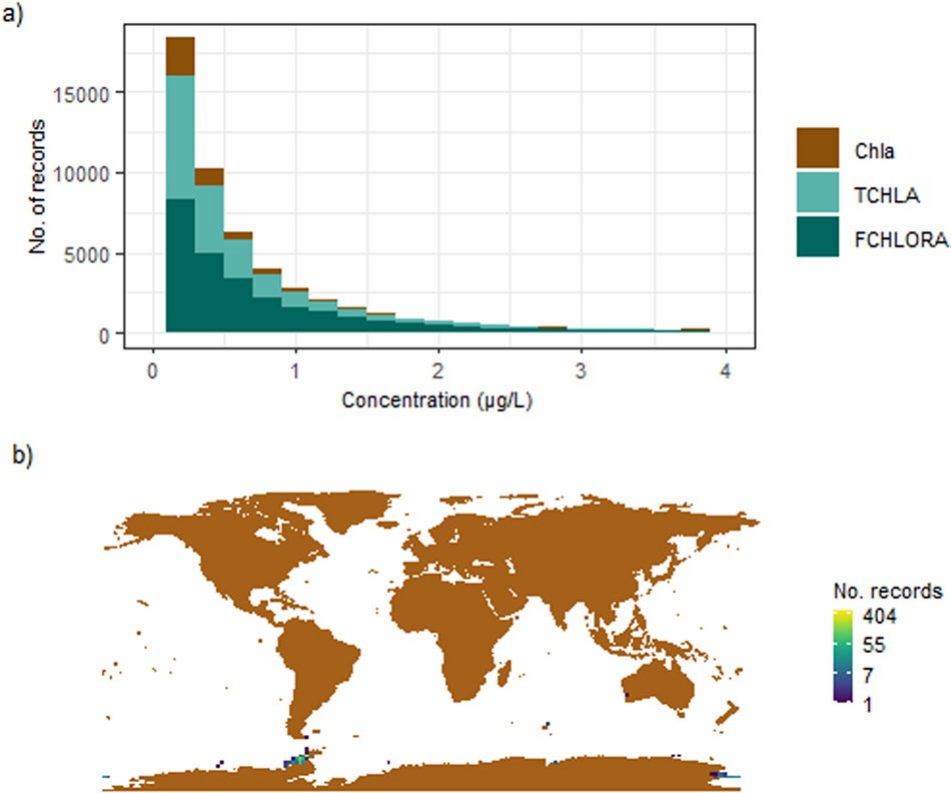
The (**a**) distribution of chlorophyll-a derived from high-performance liquid chromatography (Chla), total chlorophyll-a derived from high-performance liquid chromatography (TCHLA) and chlorophyll-a derived from fluorometric determination (FCHLORA) in the BIO-MATE data compilation and (**b**) the location of high (>10 μg/l) Chla, TCHLA and FCHLA measurements.

The biological ocean data associated with the published datasets has been interpreted correctly by the software. Overall, fluorometrically derived chlorophyll (FCHLORA), HPLC derived chlorophyll a (Chl a) and HPLC derived total chlorophyll (TCHLA) measurements show a log-normal distribution, as expected. High values (>10 μg/l) are constrained to the coastal zones as expected (Fig. 6).

**Fig. 7:**
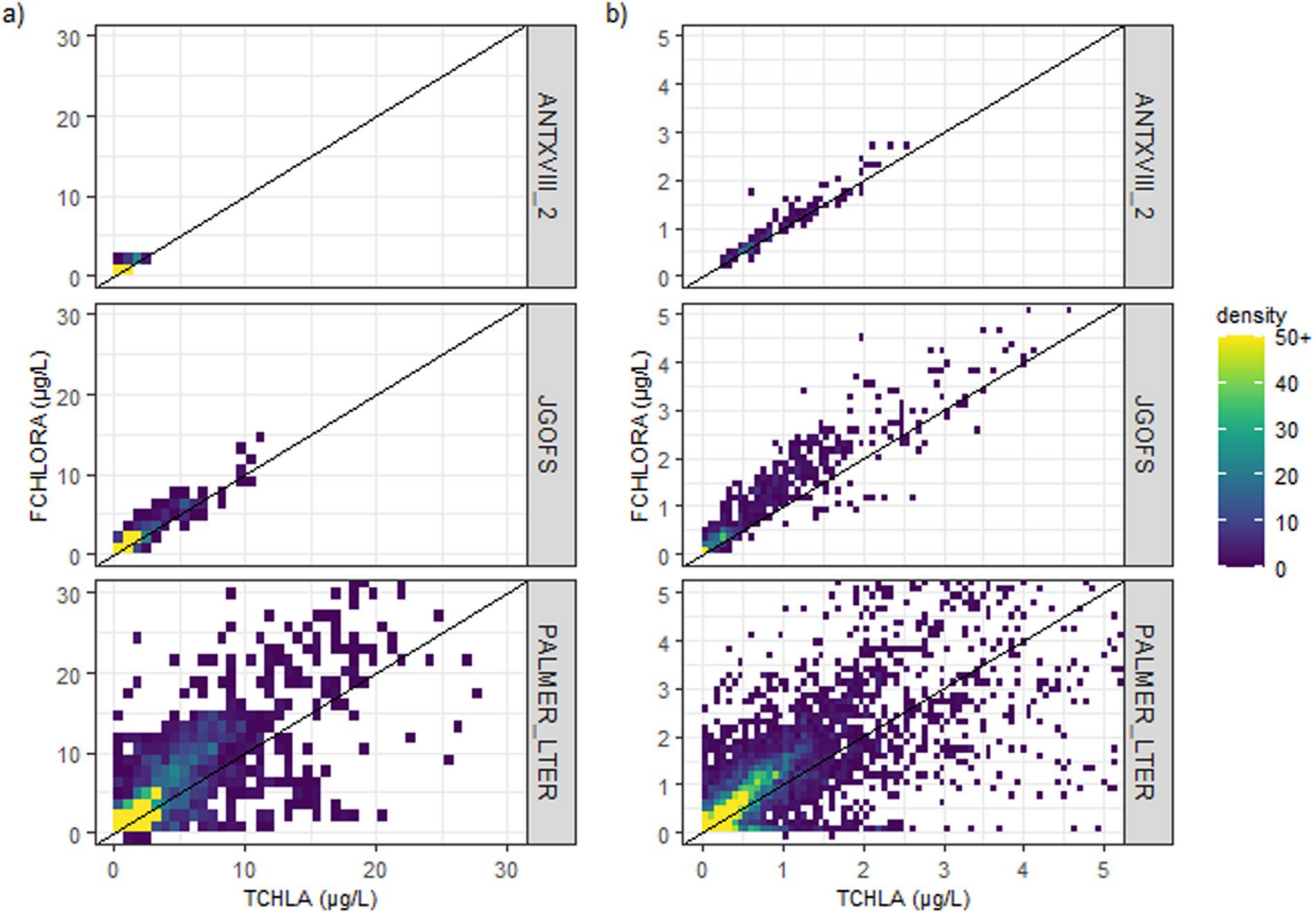
A comparison of fluorometrically derived chlorophyll (FCHLORA) methods against total chlorophyll-a derived from HPLC measurements (TCHLA). The methods presented in this figure are ANTXVII_2^491^, JGOFS^490^ and PALMER_LTER^496^ which are widely used in the dataset.

There is a linear relationship between chlorophyll-a derived from HPLC methods and chlorophyll derived from fluorometric methods (Fig. 7). Five fluorometric methods to derive chlorophyll have coincident HPLC measurements. Briefly, the ANTXVIII^492^_and JGOFS^491^ methods shows good correlation between the fluorometric and HPLC. The PALMER_LTER^497^ method shows considerable variability, which may be due to the coastal location of most samples and the influence of accessory pigments, but further investigation is needed. Only a small number of coincident HPLC measurements were collected alongside other fluorometric methods (<11), making it difficult to assess their quality.

Our validation plots show that fluorometric determination of chlorophyll tends to overestimate chlorophyll in the Southern Ocean. However, considerable variability is observed as the over-estimation or underestimation of chlorophyll-a by fluorometry is regionally dependant with changing phytoplankton assemblages^508–510^. A recent international intercomparison has also highlighted higher uncertainties, possibly due to low filtration volumes and different extraction and storage methods and suggests new standards for these measurements^511^. Despite these uncertainties, visual inspection of the profiles show that distributions of chlorophyll with depth are often well captured by fluorometric measurements.

**Fig. 8:**
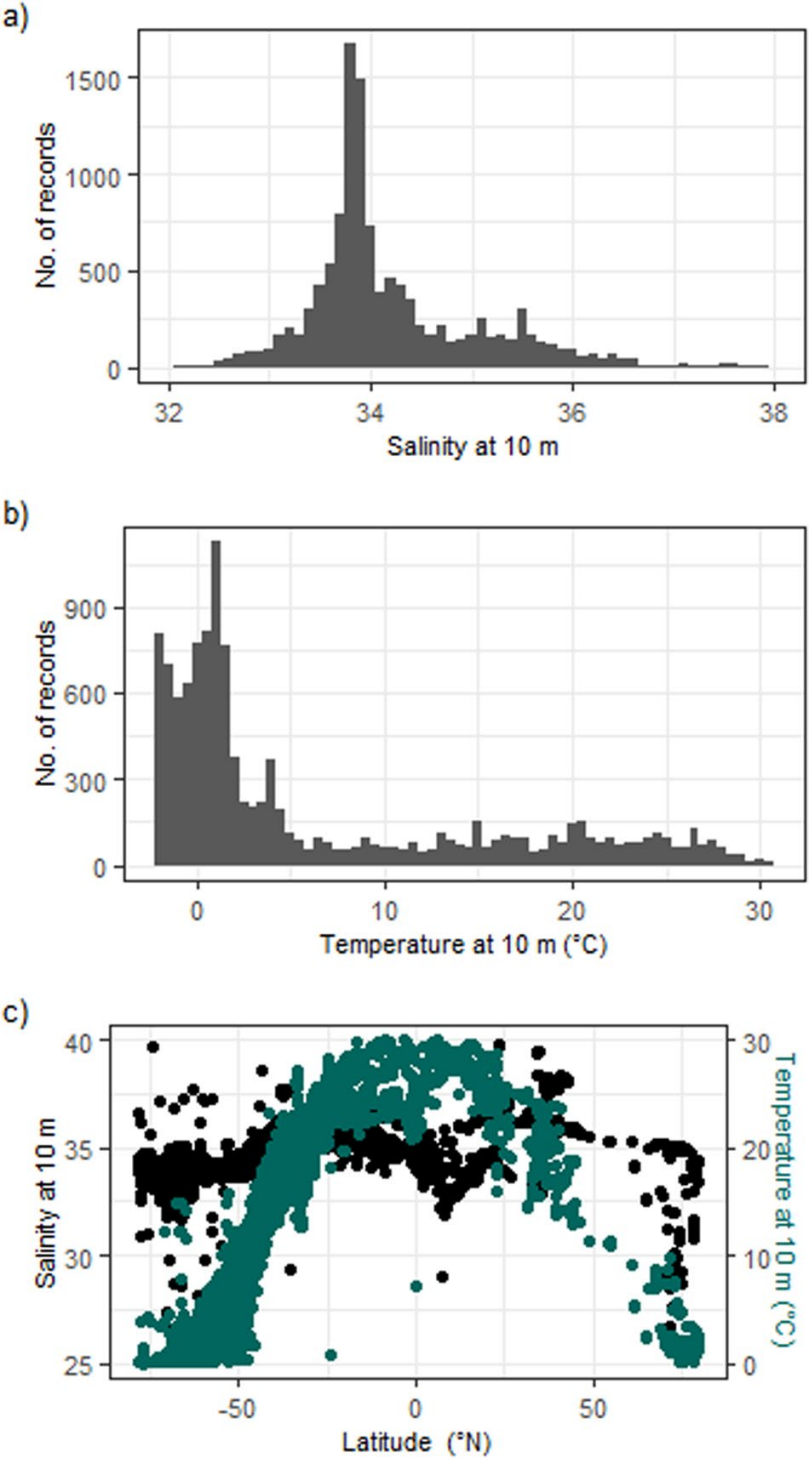
The distribution of temperature and salinity data measured at 10m by profiling sensors in the BIO-MATE data compilation.

The physical ocean data associated with published datasets has been interpreted correctly by the software. Temperature and salinity ranges fall within expected vales for the ocean and display expected trends with latitude (Fig. 8).

## Usage Notes

The community is welcome to contribute to the development of BIO-MATE software and to contribute published data to the aggregation, by following a user guide (Fig. 3).

### Contributing to BIO-MATE software development

It is recommended that changes to BIO-MATE software be made through Git Hub. Contributors can fork the existing repository (https://github.com/KimBaldry/BIOMATE-Rpackage) and make changes directly to the source code. Once changes are made, they can be directed back to the BIO-MATE R package repository and released as an updated version of the BIO-MATE software. If the BIO-MATE source code is to be significantly developed, we suggest that the corresponding author is contacted and a hand-over of the software is negotiated. We encourage the addition of new data streams to BIO-MATE, the expansion of BIO-MATE capabilities, the addition of quality assurances and increases in software efficiency.

### Contributing data to BIO-MATE

Users can create their own workflows using the BIO-MATE R package to reformat data and information (Fig. 3; https://github.com/KimBaldry/BIO-MATE/blob/main/BIO-MATE.Rmd). Once data have been reformatted, they can be submitted to the corresponding author via Git Hub (https://github.com/KimBaldry/BIOSHARE-submissions) or direct communication. We ask that all data submitted to BIO-MATE are published elsewhere and that users enter an accurate citation for the data they are submitting.

Currently, BIO-MATE only supports data files stored in delimited text formats, with structured headers and columns in a data table, and NetCDF format. The user is required to enter in some metadata to inform the software on input formats (Supplementary Processing Metadata Table).

### Recommended use in data analyses

We encourage the use of the data aggregate product as a new integrated database of biological and physical data. Data files from selected voyages can be identified using unique EXPOCODES and CTD_IDs. This makes it easy to use multiple data streams in analysis, by indexing files across these EXPOCODES. Alternatively, the selection tool on the IMAS repository helps users to select voyages using spatial bounds.

### Supplementary information

Processing Metadata

Supplementary QA/QC

Tables refered to in the Data Records Section

## Data Availability

All data processing was performed in R software (Version 3.6.3). The BIO-MATE R software is freely available (https://github.com/KimBaldry/BIOMATE-Rpackage). The semi-automated workflow and accompanying processing data used to construct the data product, along with the code used to create the data descriptor is freely accessible via Git Hub (https://github.com/KimBaldry/BIO-MATE).

## References

[1] Raymond WS (2018). The ocean’s role in climate. Oceanography.

[2] Ainley DG, Fraser WR, Smith WO, Hopkins TL, Torres JJ (1991). The structure of upper level pelagic food webs in the Antarctic: Effect of phytoplankton distribution. J. Mar. Syst..

[3] Basu S, Mackey KRM (2018). Phytoplankton as key mediators of the biological carbon pump: Their responses to a changing climate. Sustainability.

[4] Carranza MM (2018). When mixed layers are not mixed. Storm-driven mixing and bio-optical vertical gradients in mixed layers of the Southern Ocean. J. Geophys. Res. Oceans.

[5] Prairie JC, Sutherland KR, Nickols KJ, Kaltenberg AM (2012). Biophysical interactions in the plankton: A cross-scale review. Limnol. and Oceanogr. Fluids and Environments.

[6] Wihsgott JU (2019). Observations of vertical mixing in autumn and its effect on the autumn phytoplankton bloom. Prog. Oceanogr..

[7] Brody SR, Lozier MS (2015). Characterizing upper-ocean mixing and its effect on the spring phytoplankton bloom with in situ data. ICES J. Mar. Sci..

[8] Mignot A, D’Ortenzio F, Taillandier V, Cossarini G, Salon S (2019). Quantifying observational errors in biogeochemical-argo oxygen, nitrate, and chlorophyll a concentrations. Geophys. Res. Lett..

[9] Baldry K (2023). University of Tasmania.

[10] Baldry K, Strutton PG, Hill NA, Boyd PW (2024). Subsurface chlorophyll maxima reduce the performance of non-photochemical quenching corrections in the Southern Ocean. Front. Mar. Sci..

[11] Valente A (2019). A compilation of global bio-optical in situ data for ocean-colour satellite applications – version two. Earth Syst. Sci. Data.

[12] Johnson R, Strutton PG, Wright SW, McMinn A, Meiners KM (2013). Three improved satellite chlorophyll algorithms for the Southern Ocean. J. of Geophys. Res. Oceans.

[13] Sauzède R (2015). Vertical distribution of chlorophyll a concentration and phytoplankton community composition from in situ fluorescence profiles: A first database for the global ocean. Earth Syst. Sci. Data.

[14] Roesler C (2017). Recommendations for obtaining unbiased chlorophyll estimates from in situ chlorophyll fluorometers: A global analysis of WET labs ECO sensors. Limnol. and Oceanogr. Methods.

[15] Verdy A, Mazloff MR (2017). A data assimilating model for estimating Southern Ocean biogeochemistry. J. of Geophys. Res. Oceans.

[16] Rohardt G (2010). PANGAEA.

[17] Nöthig E-M, Cornils A (2019). PANGAEA.

[18] Hempel G (1985). Die expedition ANTARKTIS-III mit FS polarstern 1984/85. Reports on Polar Research.

[19] Gordon AL (2010). PANGAEA.

[20] Schnack-Schiel SB, Dieckmann GS (2004). PANGAEA.

[21] Schnack-Schiel SB (1987). The Winter-Expedition of RV Polarstern to the Antarctic ANT V/1-3. Reports on Polar Research.

[22] Hempel I, Veth C (2003). PANGAEA.

[23] Hempel I, Veth C, Smetacek V, van Beusekom J, Jacques G (2003). PANGAEA.

[24] Smetacek V, Hempel I, Schalk PH (1989). The expedition ANTARKTIS-VII/3 EPOSLEG2 of RV polarstern in 1988/89. Reports on Polar Research.

[25] Smetacek V, de Baar HJW, Bathmann U, Lochte K, Rutgers van der Loeff MM (1997). PANGAEA.

[26] Smetacek V, de Baar HJW, Bathmann U, Lochte K, Rutgers van der Loeff MM (1997). PANGAEA.

[27] Smetacek V, de Baar HJW, Bathmann U, Lochte K, Rutgers van der Loeff MM (1997). PANGAEA.

[28] Bathmann U, Smetacek V, Baar HJ, de, Fahrbach E, Krause G (1994). Die expeditionen ANTARKTIS x/6-8 des forschungsschiffes polarstern 1992/93. Reports on Polar Research.

[29] Strass VH (2010). PANGAEA.

[30] Riebesell U (2009). PANGAEA.

[31] Bathmann U, El Naggar SED, Smetacek V (2001). The expeditions ANTARKTIS XVIII/1-2 of the research vessel Polarstern in 2000. Reports on Polar Research.

[32] Smetacek V, Riebesell U (2003). PANGAEA.

[33] Peeken I, Nachtigall K (2014). PANGAEA.

[34] Smetacek V, Riebesell U (2003). PANGAEA.

[35] Smetacek V, Riebesell U (2003). PANGAEA.

[36] Smetacek V, Riebesell U (2003). PANGAEA.

[37] Smetacek V, Riebesell U (2003). PANGAEA.

[38] Smetacek V, Riebesell U (2003). PANGAEA.

[39] Smetacek V, Riebesell U (2003). PANGAEA.

[40] Smetacek V, Riebesell U (2003). PANGAEA.

[41] Strass VH (2010). PANGAEA.

[42] Smetacek V, Rohardt G (2007). PANGAEA.

[43] Bathmann U, Smetacek V, Helmke E (2005). The expeditions ANTARKTIS XXI/3-4-5 of the research vessel polarstern in 2004. Reports on Polar Research.

[44] Peeken I, Hoffmann L (2014). PANGAEA.

[45] Peeken I, Hoffmann L (2014). PANGAEA.

[46] Strass VH (2010). PANGAEA.

[47] Bathmann U, Rohardt G (2009). PANGAEA.

[48] Bathmann U (2010). The expedition of the research vessel Polarstern to the Antarctic in 2007/2008 ANT-XXIV/2. Reports on Polar Research.

[49] Peeken I, Bracher A, Murawski S (2017). PANGAEA.

[50] Schmitt B, Rohardt G (2010). PANGAEA.

[51] Macke A, Rohardt G (2009). PANGAEA.

[52] Macke A (2009). The expedition of the research vessel Polarstern to the Antarctic in 2008 ANT XXIV/4. Reports on Polar Research.

[53] Bracher A (2015). PANGAEA.

[54] Schröder M, Wisotzki A (2010). PANGAEA.

[55] Gohl K, Rohardt G (2010). PANGAEA.

[56] Gohl K (2010). The expedition of the research vessel Polarstern to the Amundsen sea, antarctica, in 2010 ANT XXVI/3. Reports on Polar Research.

[57] Peeken I, Nachtigall K (2014). PANGAEA.

[58] Peeken I, Nachtigall K (2014). PANGAEA.

[59] Rohardt G, Bracher A (2011). PANGAEA.

[60] Körtzinger A, Rohardt G (2010). PANGAEA.

[61] Körtzinger A (2011). The expedition of the research vessel Polarstern to the Antarctic in 2010 ANT XXVI/4. Reports on Polar Research.

[62] Bracher A (2015). PANGAEA.

[63] Strass VH (2016). PANGAEA.

[64] Wolf-Gladrow DA, Rohardt G (2012). PANGAEA.

[65] Wolf-Gladrow D (2013). The expedition of the research vessel Polarstern to the Antarctic in 2012 ANT XXVIII/3. Reports on Polar Research.

[66] Bracher A (2014). PANGAEA.

[67] Roca-Martí M (2015). PANGAEA.

[68] Meyer B, Rohardt G (2014). PANGAEA.

[69] Meyer B, Rohardt G (2013). PANGAEA.

[70] Meyer B, Auerswald L (2014). The expedition of the research vessel Polarstern to the Antarctic in 2013 ANT XXIX/7. Reports on Polar Research.

[71] Meyer B (2017). PANGAEA.

[72] Rohardt G, Boebel O (2017). PANGAEA.

[73] Boebel O (2017). The expedition PS103 of the research vessel Polarstern to the Weddell sea in 2016/2017. Reports on Polar Research.

[74] Boebel O, Rohardt G (2018). PANGAEA.

[75] Bracher A (2019). PANGAEA.

[76] Rohardt G, Boebel O (2020). PANGAEA.

[77] Boebel O, Rohardt G (2019). PANGAEA.

[78] Boebel O (2019). The expedition PS117 of the research vessel Polarstern to the Weddell sea in 2018/2019. Reports on Polar Research.

[79] Trull, T. RV Investigator voyage summary IN2015_V01. *CSIRO Marine National Facility* https://www.cmar.csiro.au/data/trawler/survey_list.cfm?q=&source_id=309&start=61#IN2015_V01 (2015).

[80] CSIRO. RV Investigator voyage IN2015_V01 CTD data. *CSIRO Marine National Facility* https://marlin.csiro.au/geonetwork/srv/eng/catalog.search#/metadata/1dcbfe47-d8e3-4c36-e053-08114f8cc252 (2015).

[81] CSIRO. RV Investigator voyage IN2015_V01 underway UWY data. *CSIRO Marine National Facility* https://marlin.csiro.au/geonetwork/srv/eng/catalog.search#/metadata/1160f9f9-c2b1-027f-e053-08114f8c709e (2015).

[82] Coffin, M. RV Investigator voyage summary IN2016_V01. *CSIRO Marine National Facility* https://www.cmar.csiro.au/data/trawler/survey_list.cfm?q=&source_id=309&start=51#IN2016_V01 (2016).

[83] CSIRO. RV Investigator voyage IN2016_V01 CTD data. *CSIRO Marine National Facility* https://marlin.csiro.au/geonetwork/srv/eng/catalog.search#/metadata/6ba5a0ce-b87a-4bda-b16c-3527279c3bca (2016).

[84] CSIRO. RV Investigator voyage IN2016_V01 underway UWY data. *CSIRO Marine National Facility* https://marlin.csiro.au/geonetwork/srv/eng/catalog.search#/metadata/291d1744-409c-2a7e-e053-08114f8ca4d4 (2016).

[85] Clementson, L. RV Investigator IN2016_V01, pigment and ocean colour data. *CSIRO Marine National Facility* https://catalogue-imos.aodn.org.au/geonetwork/srv/en/metadata.show?uuid=97b9fe73-ee44-437f-b2ae-5b8613f81042 (2011).

[86] Trull, T. RV Investigator voyage summary IN2016_V02. *CSIRO Marine National Facility* https://www.cmar.csiro.au/data/trawler/survey_list.cfm?q=&source_id=309&start=51#IN2016_V02 (2016).

[87] CSIRO. RV Investigator voyage IN2016_V02 CTD data. *CSIRO Marine National Facility* https://marlin.csiro.au/geonetwork/srv/eng/catalog.search#/metadata/d57331f4-3213-4817-b47e-a2ad0292148a (2016).

[88] CSIRO. RV Investigator voyage IN2016_V02 underway UWY data. *CSIRO Marine National Facility* https://marlin.csiro.au/geonetwork/srv/eng/catalog.search#/metadata/2cf37561-1c43-0d22-e053-08114f8c7cd2 (2016).

[89] Clementson, L. RV Investigator IN2016_V02, pigment and ocean colour data. *CSIRO Marine National Facility* https://catalogue-imos.aodn.org.au/geonetwork/srv/en/metadata.show?uuid=97b9fe73-ee44-437f-b2ae-5b8613f81042 (2011).

[90] Sloyan, B. RV Investigator voyage summary IN2016_V03. *CSIRO Marine National Facility* https://www.cmar.csiro.au/data/trawler/survey_list.cfm?q=&source_id=309&start=51#IN2016_V02 (2016).

[91] CSIRO. RV Investigator voyage IN2016_V03 CTD data. *CSIRO Marine National Facility* https://marlin.csiro.au/geonetwork/srv/eng/catalog.search#/metadata/d57331f4-3213-4817-b47e-a2ad0292148a (2016).

[92] CSIRO. RV Investigator voyage IN2016_V03 underway UWY data. *CSIRO Marine National Facility* https://marlin.csiro.au/geonetwork/srv/eng/catalog.search#/metadata/2cf37561-1c43-0d22-e053-08114f8c7cd2 (2016).

[93] Sloyan, B. M. *et al*. Dissolved inorganic carbon (DIC), total alkalinity, pH on total scale, and other hydrographic and chemical data obtained during the SOCCOM float deployment expedition onboard r/v investigator cruise IN2016_V03 along the GO-SHIP section P15S (EXPOCODE 096U20160426) from 2016-04-26 to 2016-06-30 (NCEI accession 0168141). *National Oceanic and Atmospheric Administration, Department of Commerce* https://doi.org/v10.7289/v5p26wdd (2017).

[94] Rintoul, S. RV Investigator voyage summary IN2018_V01. *CSIRO Marine National Facility* https://www.cmar.csiro.au/data/trawler/survey_list.cfm?q=&source_id=309&start=51#IN2016_V02 (2018).

[95] CSIRO. RV Investigator voyage IN2018_V01 CTD data. *CSIRO Marine National Facility* https://marlin.csiro.au/geonetwork/srv/eng/catalog.search#/metadata/d57331f4-3213-4817-b47e-a2ad0292148a (2018).

[96] CSIRO. RV Investigator voyage IN2018_V01 underway UWY data. *CSIRO Marine National Facility* https://marlin.csiro.au/geonetwork/srv/eng/catalog.search#/metadata/2cf37561-1c43-0d22-e053-08114f8c7cd2 (2018).

[97] Clementson, L. RV Investigator IN2016_V03, pigment and ocean colour data. *CSIRO Marine National Facility* https://portal.aodn.org.au/search?uuid=97b9fe73-ee44-437f-b2ae-5b8613f81042 (2020).

[98] Rosenberg M (2002). Australian Antarctic Data Centre.

[99] Wright S (2013). Australian Antarctic Data Centre.

[100] Reeve, J. Aurora Australis voyage 6 (AAMBER2) 1990-91 underway data. *Australian Antarctic Data Centre* https://data.aad.gov.au/metadata/records/199091060 (1999).

[101] Vaudrey DJ (2001). Australian Antarctic Data Centre.

[102] Rosenberg M, Ronai B (2001). Australian Antarctic Data Centre.

[103] Wright S (2013). Australian Antarctic Data Centre.

[104] Reeve, J. Aurora Australis voyage 1 and 1.1 (WOCE91) 1991-92 underway data. *Australian Antarctic Data Centre* https://data.aad.gov.au/metadata/records/199192010 (1999).

[105] Wright S (2013). Australian Antarctic Data Centre.

[106] Reeve, J. Aurora Australis voyage 4 1991-92 underway data. *Australian Antarctic Data Centre* https://data.aad.gov.au/metadata/records/199192040 (1999).

[107] Hosie G, Watts D (2018). Australian Antarctic Data Centre.

[108] Wright S (2013). Australian Antarctic Data Centre.

[109] Reeve, J. Aurora Australis voyage 7 (KROCK) 1992-93 underway data. *Australian Antarctic Data Centre* https://data.aad.gov.au/metadata/records/199293070 (1999).

[110] Mark Rosenberg RE, Rintoul S (2001). Australian Antarctic Data Centre.

[111] Rosenberg M (2001). Australian Antarctic Data Centre.

[112] Wright, S. Aurora Australis voyage 9 (WOES) 1992-93 chlorophyll a data. *Australian Antarctic Data Centre* https://data.aad.gov.au/metadata/records/199293090 (2013).

[113] Reeve, J. Aurora Australis voyages 9 and 9.1 (WOES and WORSE) 1992-93 underway data. *Australian Antarctic Data Centre*, https://data.aad.gov.au/metadata/records/199293090 (1999).

[114] Wright, S. The role of antarctic marine protists in trophodynamics and global change and the impact of UV-B on these organisms. *Australian Antarctic Data Centre* https://data.aad.gov.au/metadata/records/ASAC_40 (2013).

[115] Reeve, J. Aurora Australis voyage 4 1993-94 underway data. *Australian Antarctic Data Centre* https://data.aad.gov.au/metadata/records/199394040 (1999).

[116] Rosenberg M (2001). Australian Antarctic Data Centre.

[117] Rosenberg M (2001). Australian Antarctic Data Centre.

[118] Wright S (2004). Australian Antarctic Data Centre.

[119] Reeve, J. Aurora Australis voyage 7 (SHAM) 1993-94 underway data. *Australian Antarctic Data Centre* https://data.aad.gov.au/metadata/records/199394070 (1999).

[120] Rosenberg M, Eriksen R, Bell S, Rintoul S (2001). Australian Antarctic Data Centre.

[121] Rosenberg M (2001). Australian Antarctic Data Centre.

[122] Reeve, J. Aurora Australis voyage 4 (WOCET) 1994-95 underway data. *Australian Antarctic Data Centre* https://data.aad.gov.au/metadata/records/199495040 (1999).

[123] Rosenberg M (2001). Australian Antarctic Data Centre.

[124] Rosenberg M (2001). Australian Antarctic Data Centre.

[125] Wright S (2004). Australian Antarctic Data Centre.

[126] Reeve, J. Aurora Australis voyage 1 (ABSTAIN) 1995-96 underway data. *Australian Antarctic Data Centre* https://data.aad.gov.au/metadata/records/199596010 (1999).

[127] Rosenberg M (2008). Australian Antarctic Data Centre.

[128] Steve Rintoul MR, Bindoff N (2008). Australian Antarctic Data Centre.

[129] Wright S (2010). Australian Antarctic Data Centre.

[130] Reeve, J. Aurora Australis voyage 4 (BROKE) 1995-96 underway data. *Australian Antarctic Data Centre* https://data.aad.gov.au/metadata/records/199596040/ (1999).

[131] Rintoul S, Rosenberg M, Trull T (2001). Australian Antarctic Data Centre.

[132] Wright S (2011). Australian Antarctic Data Centre.

[133] Reeve, J. Aurora Australis voyage 6 (SNARK) 1997-98 underway data. *Australian Antarctic Data Centre* https://data.aad.gov.au/metadata/records/199798060 (1999).

[134] Rosenberg M (2004). Australian Antarctic Data Centre.

[135] Rosenberg M, Bindoff N, Williams G (2004). Australian Antarctic Data Centre.

[136] Wright S, van den Enden D (2012). Australian Antarctic Data Centre.

[137] Reeve, J. Aurora Australis voyage 1 (IDIOTS) 1999-00 underway data. *Australian Antarctic Data Centre* https://data.aad.gov.au/metadata/records/199900010 (1999).

[138] Rosenberg M (2020). Australian Antarctic Data Centre.

[139] Wright S (2013). Australian Antarctic Data Centre.

[140] Reeve, J. Aurora Australis Voyage 6 2000-2001 underway data. *Australian Antarctic Data Centre* https://data.aad.gov.au/metadata/records/200001060 (2001).

[141] Rosenberg M, Rintoul S, Bray S, Moy C, Johnston N (2004). Australian Antarctic Data Centre.

[142] Rintoul S, Rosenberg M (2004). Australian Antarctic Data Centre.

[143] Reeve, J. Aurora Australis voyage 3 2001-2002 underway data. *Australian Antarctic Data Centre* https://data.aad.gov.au/metadata/records/200102030 (2001).

[144] Rosenberg M (2020). Australian Antarctic Data Centre.

[145] Rosenberg M (2004). Australian Antarctic Data Centre.

[146] Rintoul S, Rosenberg M, Bindoff N, Church J, Yasushi F (2004). Australian Antarctic Data Centre.

[147] Wright S (2013). Australian Antarctic Data Centre.

[148] Reeve, J. Aurora Australis voyage 4 2002-2003 underway data. *Australian Antarctic Data Centre* https://data.aad.gov.au/metadata/records/200203040 (2003).

[149] Rosenberg M (2004). Australian Antarctic Data Centre.

[150] Rosenberg M, Ronai B (2005). Australian Antarctic Data Centre.

[151] Wright S (2013). Australian Antarctic Data Centre.

[152] Reeve, J. Aurora Australis voyage 4 2003-2004 underway data. *Australian Antarctic Data Centre* https://data.aad.gov.au/metadata/records/200304040 (2004).

[153] Wright S (2013). Australian Antarctic Data Centre.

[154] Wiley P (2005). Australian Antarctic Data Centre.

[155] Rosenberg M, Rintoul S, Fukamachi M, Church J (2006). Australian Antarctic Data Centre.

[156] Rosenberg M (2019). Australian Antarctic Data Centre.

[157] Rosenberg M, Gorton R (2019). Australian Antarctic Data Centre.

[158] Wright S (2010). Australian Antarctic Data Centre.

[159] Wiley, P. Aurora Australis voyage 3 2005-2006 (BROKE-West) underway data. *Australian Antarctic Data Centre* https://data.aad.gov.au/metadata/records/200506030 (2006).

[160] Rosenberg M (2010). Australian Antarctic Data Centre.

[161] Mark Rosenberg WRH, Griffiths B (2010). Australian Antarctic Data Centre.

[162] Howard W, Wright S, Griffiths B (2014). Australian Antarctic Data Centre.

[163] Jono Reeve, W. R. H. & Griffiths, B. Aurora Australis voyage V3 2006/07 (SAZ-SENSE) track and underway data. *Australian Antarctic Data Centre* https://data.aad.gov.au/metadata/records/200607030 (2009).

[164] Rosenberg M, Rintoul S (2019). Australian Antarctic Data Centre.

[165] Rosenberg M, Rintoul S (2019). Australian Antarctic Data Centre.

[166] Wright S (2013). Australian Antarctic Data Centre.

[167] Rintoul, S. R., van Wijk, E. M. Aurora australis southern ocean oceanographic data, cruise au1121 2010/11 VMS. *Australian Antarctic Data Centre* https://data.aad.gov.au/metadata/records/au1121 (2014).

[168] Steve Rintoul MR (2013). Australian Antarctic Data Centre.

[169] Rosenberg M, Eriksen R (2019). Australian Antarctic Data Centre.

[170] Bestley S, Rosenberg M (2019). Australian Antarctic Data Centre.

[171] Symmons, L. Aurora Australis voyage 3 2015/16 track and underway data. *Australian Antarctic Data Centre* https://data.aad.gov.au/metadata/records/201516030 (2016).

[172] CSIRO. Franklin voyage FR 10/97 CTD data. *CSIRO Marine National Facility* https://www.marine.csiro.au/data/trawler/dataset.cfm?survey=FR199710&data_type=ctd (1999).

[173] Clementson, L. Franklin cruise 10/97 HPLC pigment and ocean colour data. *CSIRO Marine National Facility* https://portal.aodn.org.au/search?uuid=97b9fe73-ee44-437f-b2ae-5b8613f81042 (2020).

[174] Parslow J (1995). Cruise report SS 01/95: January 14 - February 2, 1995. CSIRO Marine National Facility.

[175] CSIRO. Southern Surveyor voyage SS 01/95 CTD data. *CSIRO Marine National Facility* https://marlin.csiro.au/geonetwork/srv/eng/catalog.search;jsessionid=fwib9h30l5g11n86sk9ouv655#/metadata/7780aa58-6434-453b-9bf6-b8ba0bb93533 (2014).

[176] Clementson, L. Southern Surveyor SS01/95 HPLC pigment and ocean colour data. *Australian Ocean Data Network* https://portal.aodn.org.au/search?uuid=97b9fe73-ee44-437f-b2ae-5b8613f81042 (2020).

[177] Tillbrook, B. SS 11/95 Survey details and related metadata, reports, events and data. *CSIRO Marine National Facility* https://www.cmar.csiro.au/data/trawler/survey_list.cfm?q=&source_id=8&start=161#SS199511 (1995).

[178] CSIRO. Southern Surveyor voyage SS 11/95 CTD data. *CSIRO Marine National Facility* https://marlin.csiro.au/geonetwork/srv/eng/catalog.search#/metadata/005cb0d3-c96e-4124-8852-19fa741b633a (1998).

[179] Clementson, L. Southern Surveyor voyage SS 11/95 HPLC pigment and ocean colour data. *Australian Ocean Data Network* https://portal.aodn.org.au/search?uuid=97b9fe73-ee44-437f-b2ae-5b8613f81042 (2020).

[180] CSIRO. Southern Surveyor voyage SS 11/95 underway data. *CSIRO Marine National Facility* https://marlin.csiro.au/geonetwork/srv/eng/catalog.search#/metadata/8121b9d8-cefb-4fec-a6a3-1659bfab00dc (2001).

[181] Williams, A. & Bax, N. SS 02/96 Survey details and related metadata, reports, events and data. *CSIRO Marine National Facility* https://www.cmar.csiro.au/data/trawler/survey_list.cfm?q=&source_id=8&start=161#SS199511 (1996).

[182] CSIRO. Southern Surveyor voyage SS 02/96 CTD data. *CSIRO Marine National Facility* https://marlin.csiro.au/geonetwork/srv/eng/catalog.search#/metadata/3463b550-9abc-4816-8d39-9dfd13a700ed (2001).

[183] Clementson, L. Southern Surveyor voyage SS 02/96 HPLC pigment and ocean colour data. *CSIRO Marine National Facility* https://portal.aodn.org.au/search?uuid=97b9fe73-ee44-437f-b2ae-5b8613f81042 (2020).

[184] CSIRO. Southern Surveyor voyage SS 02/96 underway Data. *CSIRO Marine National Facility* https://marlin.csiro.au/geonetwork/srv/eng/catalog.search#/metadata/cfc98f1a-498e-43d1-a732-f4c6b1a8eb38 (2001).

[185] Young, J. SS 03/96 survey details and related metadata, reports, events and data. *CSIRO Marine National Facility* https://www.cmar.csiro.au/data/trawler/survey_details.cfm?survey=SS199603 (1996).

[186] CSIRO. Southern Surveyor voyage SS 03/96 CTD data. *CSIRO Marine National Facility* https://marlin.csiro.au/geonetwork/srv/eng/catalog.search#/metadata/900e2c7e-b0d2-4740-a458-8503a5340de7 (1998).

[187] Clementson, L. Southern Surveyor voyage SS 03/96 HPLC pigment and ocean colour data. *Australian Ocean Data Network* https://portal.aodn.org.au/search?uuid=97b9fe73-ee44-437f-b2ae-5b8613f81042 (2020).

[188] Williams, A. & Liron, C. Cruise report SS 06/96 November 20 - December 19, 1996. 10.4225/08/586fdc688f7a9 (1996).

[189] CSIRO. Southern Surveyor voyage SS 06/96 CTD data. *CSIRO Marine National Facility* https://marlin.csiro.au/geonetwork/srv/eng/catalog.search#/metadata/abc685f6-528a-4c18-b2b0-38f197e010c5 (1996).

[190] CSIRO. Southern Surveyor voyage SS 06/96 underway data. *CSIRO Marine National Facility* https://marlin.csiro.au/geonetwork/srv/eng/catalog.search#/metadata/4578cb46-47e4-4d2e-abd0-0fe631e96f2b (1996).

[191] Clementson, L. Southern Surveyor voyage SS 06/96 HPLC pigment and ocean colour data. *Australian Ocean Data Network* https://portal.aodn.org.au/search?uuid=97b9fe73-ee44-437f-b2ae-5b8613f81042 (2020).

[192] Koslow, T. & Kloser, R. Cruise report SS 01/99: January 10 - February 4, 1999. 10.4225/08/58712d755cf79 (1996).

[193] Clementson, L. Southern Surveyor voyage SS 01/96 HPLC pigment and ocean colour data. *Australian Ocean Data Network* https://portal.aodn.org.au/search?uuid=97b9fe73-ee44-437f-b2ae-5b8613f81042 (2020).

[194] CSIRO. Southern Surveyor voyage SS 02/99 CTD data. *CSIRO Marine National Facility* https://marlin.csiro.au/geonetwork/srv/eng/catalog.search#/metadata/c7052a7f-705d-4fb5-95b1-142888b30430 (2004).

[195] Clementson, L. Southern Surveyor voyage SS 02/99 HPLC pigment and ocean colour data. *Australian Ocean Data Network* https://portal.aodn.org.au/search?uuid=97b9fe73-ee44-437f-b2ae-5b8613f81042 (2020).

[196] CSIRO. Southern Surveyor voyage SS 02/99 underway data. *CSIRO Marine National Facility* https://marlin.csiro.au/geonetwork/srv/eng/catalog.search#/metadata/a69eeaef-844c-45d4-a3ee-09a7d771665d (2004).

[197] Koslow, T. Voyage summary SS07/2003. *CSIRO Marine National Facility* https://www.cmar.csiro.au/data/trawler/survey_details.cfm?survey=SS200307 (2003).

[198] CSIRO. Southern Surveyor voyage SS 07/2003 underway data. *CSIRO Marine National Facility* https://marlin.csiro.au/geonetwork/srv/eng/catalog.search#/metadata/97576cdc-fe47-4f0a-81b2-8595b73006e9 (2007).

[199] Clementson, L. SRFME ocean colour data - offShore, Southern Surveyor SS 07/2003. *Australian Ocean Data Network* https://portal.aodn.org.au/search?uuid=97b9fe73-ee44-437f-b2ae-5b8613f81042 (2020).

[200] Koslow, T. Voyage summary SS01/2004. *CSIRO Marine National Facility* https://www.cmar.csiro.au/data/trawler/survey_details.cfm?survey=SS200401 (2004).

[201] CSIRO. Southern Surveyor voyage SS 01/2004 CTD data. *CSIRO Marine National Facility* https://marlin.csiro.au/geonetwork/srv/eng/catalog.search#/metadata/dc42f374-b762-4bea-956a-f44a6cda956e (2000).

[202] Clementson, L. SRFME ocean colour data - offShore, Southern Surveyor SS 01/2004. *Australian Ocean Data Network* https://portal.aodn.org.au/search?uuid=97b9fe73-ee44-437f-b2ae-5b8613f81042 (2020).

[203] CSIRO. Southern Surveyor voyage SS 01/2004 underway data. *CSIRO Marine National Facility* https://marlin.csiro.au/geonetwork/srv/eng/catalog.search#/metadata/f82466ce-fbd7-4986-8008-a828c8747189 (2011).

[204] Kloser, R. Voyage summary SS 07/2004. *CSIRO Marine National Facility* https://www.cmar.csiro.au/data/trawler/survey_details.cfm?survey=SS200407 (2004).

[205] CSIRO. Southern Surveyor voyage SS 07/2004 CTD data. *CSIRO Marine National Facility* https://marlin.csiro.au/geonetwork/srv/eng/catalog.search#/metadata/5811196d-970a-4415-9df6-db9e75ecc58e (2007).

[206] Clementson, L. Southern Surveyor voyage SS 07/2004 HPLC pigment and ocean colour data. *Australian Ocean Data Network* https://portal.aodn.org.au/search?uuid=97b9fe73-ee44-437f-b2ae-5b8613f81042 (2020).

[207] CSIRO. Southern Surveyor voyage SS 07/2004 underway data. *CSIRO Marine National Facility* https://marlin.csiro.au/geonetwork/srv/eng/catalog.search#/metadata/7c6f72fb-88e7-4cc5-a83b-e8ae9b98ea00 (2007).

[208] Alan Williams, R. K. & Bax, N. Voyage summary SS07/2005. *CSIRO Marine National Facility* https://www.cmar.csiro.au/data/trawler/survey_details.cfm?survey=SS200401 (2004).

[209] CSIRO. Southern Surveyor voyage SS 07/2005 CTD data. *CSIRO Marine National Facility* https://marlin.csiro.au/geonetwork/srv/eng/catalog.search#/metadata/d93be039-6534-4f53-a7c9-b0c9cc991699 (2008).

[210] Clementson, L. Southern Surveyor voyage SS 07/2005 HPLC pigment and ocean colour Data. *Australian Ocean Data Network* https://portal.aodn.org.au/search?uuid=97b9fe73-ee44-437f-b2ae-5b8613f81042 (2020).

[211] CSIRO. Southern Surveyor voyage SS 07/2005 underway data. *CSIRO Marine National Facility* https://marlin.csiro.au/geonetwork/srv/eng/catalog.search#/metadata/8ead389a-489f-4ad1-ad08-9a4675b3712b (2008).

[212] Williams, A. & Kloser, R. Voyage summary SS07/2005. *CSIRO Marine National Facility* https://www.cmar.csiro.au/data/trawler/survey_details.cfm?survey=SS200510 (2004).

[213] CSIRO. Southern Surveyor voyage SS 10/2005 CTD data. *CSIRO Marine National Facility* https://marlin.csiro.au/geonetwork/srv/eng/catalog.search#/metadata/0c369d29-6063-4cce-9d58-61ec8c6b4f0f (2006).

[214] Clementson, L. Southern Surveyor voyage SS 10/2005, pigment and ocean colour data. *Australian Ocean Data Network* https://portal.aodn.org.au/search?uuid=97b9fe73-ee44-437f-b2ae-5b8613f81042 (2020).

[215] CSIRO. Southern Surveyor voyage SS 10/2005 underway data. *CSIRO Marine National Facility* https://marlin.csiro.au/geonetwork/srv/eng/catalog.search#/metadata/52a34094-58c0-4dd0-987e-b821739db500 (2007).

[216] Trull, T. SS 03/2006. Voyage details and related metadata, reports, events and data. *CSIRO Marine National Facility* https://www.cmar.csiro.au/data/trawler/survey_details.cfm?survey=SS200603 (2006).

[217] Brodie, P. Southern Surveyor voyage SS 03/2006 CTD data. *CSIRO Marine National Facility* https://marlin.csiro.au/geonetwork/srv/eng/catalog.search#/metadata/e09df8fb-ec43-4495-a64e-9bd5bd3a0f4d (2011).

[218] Clementson, L. Southern Surveyor voyage SS 03/2006, pigment and ocean colour data. https://portal.aodn.org.au/search?uuid=97b9fe73-ee44-437f-b2ae-5b8613f81042 (2020).

[219] CSIRO. Southern Surveyor voyage SS 03/2006 underway data. *CSIRO Marine National Facility* https://marlin.csiro.au/geonetwork/srv/eng/catalog.search#/metadata/46c96e26-0064-406c-a1ec-b3bec4103940 (2011).

[220] Williams, A. & Kloser, R. Voyage summary SS2010_v06. *CSIRO Marine National Facility* https://www.cmar.csiro.au/data/trawler/survey_details.cfm?survey=SS2010_V06 (2010).

[221] CSIRO. Southern Surveyor voyage SS2010_V06 CTD data. *CSIRO Marine National Facility* https://marlin.csiro.au/geonetwork/srv/eng/catalog.search#/metadata/bc1b3741-e742-5039-e044-00144f7bc0f4 (2012).

[222] Clementson, L. Southern Surveyor voyage SS2010_v06 HPLC pigment data. *Australian Ocean Data Network* https://portal.aodn.org.au/search?uuid=97b9fe73-ee44-437f-b2ae-5b8613f81042 (2020).

[223] CSIRO. Southern Surveyor voyage SS2010_V06 underway data. *CSIRO Marine National Facility* https://marlin.csiro.au/geonetwork/srv/eng/catalog.search#/metadata/bc1b3741-e7ef-5039-e044-00144f7bc0f4 (2011).

[224] Doblin, M. Voyage summary SS2010_V09. *CSIRO Marine National Facility* https://www.cmar.csiro.au/data/trawler/survey_details.cfm?survey=SS2010_V09 (2010).

[225] CSIRO. Southern Surveyor voyage SS2010_V09 CTD data. *CSIRO Marine National Facility* https://marlin.csiro.au/geonetwork/srv/eng/catalog.search#/metadata/bc1b3741-e7d9-5039-e044-00144f7bc0f4 (2012).

[226] Clementson, L. Southern Surveyor voyage SS2010_V09 HPLC pigment and ocean colour data. *Australian Ocean Data Network* https://portal.aodn.org.au/search?uuid=97b9fe73-ee44-437f-b2ae-5b8613f81042 (2020).

[227] CSIRO. Southern Surveyor voyage SS2010_V09 underway data. *CSIRO Marine National Facility* https://marlin.csiro.au/geonetwork/srv/eng/catalog.search#/metadata/bc1b3741-e78a-5039-e044-00144f7bc0f4 (2010).

[228] Phillips, H. VOYAGE SUMMARY SS2012_V04. *CSIRO Marine National Facility* https://www.cmar.csiro.au/data/trawler/survey_details.cfm?survey=SS2012_V04 (2010).

[229] CSIRO. Southern Surveyor voyage SS2012_V04 CTD data. *CSIRO Marine National Facility* https://marlin.csiro.au/geonetwork/srv/eng/catalog.search#/metadata/ccb13735-44d1-3ec9-e043-08114f8c29d9 (2012).

[230] Clementson, L. Southern Surveyor voyage SS2012_V04 pigment and ocean colour data. *Australian Ocean Data Network* https://portal.aodn.org.au/search?uuid=97b9fe73-ee44-437f-b2ae-5b8613f81042 (2020).

[231] CSIRO. Southern Surveyor voyage SS2012_V04 underway data. *CSIRO Marine National Facility* https://marlin.csiro.au/geonetwork/srv/eng/catalog.search#/metadata/dc065655-d9f8-4782-e043-08114f8c8963 (2012).

[232] Wilcox, C. Voyage summary SS2012_T06. *CSIRO Marine National Facility* https://www.cmar.csiro.au/data/trawler/survey_details.cfm?survey=SS2012_T06 (2012).

[233] CSIRO. Southern Surveyor voyage SS2012_T06 CTD data. *CSIRO Marine National Facility* https://marlin.csiro.au/geonetwork/srv/eng/catalog.search#/metadata/d864efad-75a4-0745-e043-08114f8ca335 (2013).

[234] Clementson, L. Southern Surveyor voyage SS2012_T06 pigment and ocean colour data. *Australian Ocean Data Network* https://portal.aodn.org.au/search?uuid=97b9fe73-ee44-437f-b2ae-5b8613f81042 (2020).

[235] CSIRO. Southern Surveyor voyage SS2012_T06 underway data. *CSIRO Marine National Facility* https://marlin.csiro.au/geonetwork/srv/eng/catalog.search#/metadata/dc075527-6b13-26ae-e043-08114f8c5f4c (2012).

[236] Trull, T. SS2013_V04. Voyage details and related metadata, reports, events and data. *CSIRO Marine National Facility* https://www.cmar.csiro.au/data/trawler/survey_details.cfm?survey=SS2013_V04 (2013).

[237] Kippo, H. Southern Surveyor voyage SS2013_V04 CTD data. *CSIRO Marine National Facility* https://marlin.csiro.au/geonetwork/srv/eng/catalog.search#/metadata/ed3ab483-5373-2bf5-e043-08114f8cf4f8 (2013).

[238] Clementson, L. Southern Surveyor voyage SS2013_v04, pigment and ocean colour data. *Australian Ocean Data Network* https://portal.aodn.org.au/search?uuid=97b9fe73-ee44-437f-b2ae-5b8613f81042 (2020).

[239] CSIRO. Southern Surveyor voyage SS2013_V04 underway data. *CSIRO Marine National Facility* https://marlin.csiro.au/geonetwork/srv/eng/catalog.search#/metadata/f4fed110-7324-268e-e043-08114f8c160 (2011).

[240] García MA (2002). PANGAEA.

[241] Ricardo Anadón, M. E. pH, alkalinity, temperature, salinity and other variables collected from discrete sample and profile observations using CTD, bottle and other instruments from the HESPERIDES in the south Atlantic Ocean from 1995-12-03 to 1996-01-05 (NCEI accession 0113549). *NCEI*10.3334/cdiac/otg.carina_29he19951203 (2013).

[242] García MA (2002). PANGAEA.

[243] Ricardo Anadón, M. E. pH, alkalinity, temperature, salinity and other variables collected from discrete sample and profile observations using CTD, bottle and other instruments from the HESPERIDES in the south Atlantic Ocean from 1996-01-17 to 1996-02-05 (NCEI accession 0113755). https://doi.org/110.3334/CDIAC/otg.CARINA_29HE19960117 (2013).

[244] Johnson, G. C. Documentation from cruise 31DSCG94_1. *CCHDO* https://cchdo.ucsd.edu/cruise/31DSCG94_1 (2015).

[245] Johnson, G. C. CTD data from cruise 31DSCG94_1. *CCHDO* https://cchdo.ucsd.edu/cruise/31DSCG94_1 (2015).

[246] Feely, R. A., M. R., Bullister, J. L. Dissolved inorganic carbon, pH, alkalinity, temperature, salinity and other variables collected from discrete sample and profile observations using CTD, bottle and other instruments from NOAA ship Discoverer in the Gulf of Alaska and north Pacific Ocean from 1991-03-07 to 1991-04-07 (NCEI accession 0115175). *NCEI*10.3334/cdiac/otg.31dicgc91_2 (2013).

[247] Smith, W. Directory of data in /jgofs/southern/nbp96_4A/. *USJGOFS* http://usjgofs.whoi.edu/jg/dir/jgofs/southern/nbp96_4A/ (2005).

[248] Morrison, J. Final version CTD, including beam attenuation optics. *USJGOFS* http://usjgofs.whoi.edu/jg/dir/jgofs/southern/nbp96_4A/ (2005).

[249] Anderson, B. shipboard underway data. *USJGOFS* http://usjgofs.whoi.edu/jg/dir/jgofs/southern/nbp96_4A/ (2005).

[250] Smith, W. O. Fluorometric chlorophyll-a & phaeopigments. *USJGOFS* http://usjgofs.whoi.edu/jg/dir/jgofs/southern/nbp96_4A/ (2005).

[251] Bidigare, R. R. Pigments, HPLC method, sampled from bottle and TM casts. *USJGOFS* http://usjgofs.whoi.edu/jg/dir/jgofs/southern/nbp96_4A/ (2005).

[252] Smith, W. O. Particulate organic carbon, nitrogen from CTD casts. *USJGOFS* http://usjgofs.whoi.edu/jg/dir/jgofs/southern/nbp96_4A/ (2005).

[253] Marra, J. Directory of data in /jgofs/southern/nbp97_1/. *USJGOFS* http://usjgofs.whoi.edu/jg/dir/jgofs/southern/nbp97_1/ (2005).

[254] Morrison, J. Final version CTD, including beam attenuation optics. *USJGOFS* http://usjgofs.whoi.edu/jg/dir/jgofs/southern/nbp97_1/ (2005).

[255] Anderson, B. Shipboard underway data. *USJGOFS* http://usjgofs.whoi.edu/jg/dir/jgofs/southern/nbp97_1/ (2005).

[256] Smith, W. O. Fluorometric chlorophyll-a & phaeopigments. *USJGOFS* http://usjgofs.whoi.edu/jg/dir/jgofs/southern/nbp97_1/ (2005).

[257] Bidigare, R. R. Pigments, HPLC method, sampled from bottle and TM casts. *USJGOFS* http://usjgofs.whoi.edu/jg/dir/jgofs/southern/nbp97_1/ (2005).

[258] Smith, W. O. Particulate organic carbon, nitrogen from CTD casts. *USJGOFS* http://usjgofs.whoi.edu/jg/dir/jgofs/southern/nbp97_1/ (2005).

[259] Ducklow, H. Directory of data in /jgofs/southern/nbp97_3/. *USJGOFS* http://usjgofs.whoi.edu/jg/dir/jgofs/southern/nbp97_3/ (2005).

[260] Morrison, J. Final version CTD, including beam attenuation optics. *USJGOFS* http://usjgofs.whoi.edu/jg/dir/jgofs/southern/nbp97_3/ (2005).

[261] Anderson, B. Shipboard underway data. *USJGOFS* http://usjgofs.whoi.edu/jg/dir/jgofs/southern/nbp97_3/ (2005).

[262] Smith, W. O. Fluorometric chlorophyll-a & phaeopigments. *USJGOFS* http://usjgofs.whoi.edu/jg/dir/jgofs/southern/nbp97_3/ (2005).

[263] Bidigare, R. R. Pigments, HPLC method, sampled from bottle and TM casts. *USJGOFS* http://usjgofs.whoi.edu/jg/dir/jgofs/southern/nbp97_3/ (2005).

[264] Smith, W. O. Particulate organic carbon, nitrogen from CTD casts. *USJGOFS* http://usjgofs.whoi.edu/jg/dir/jgofs/southern/nbp97_3/ (2005).

[265] Smith, W. Directory of data in /jgofs/southern/nbp97_8/. *USJGOFS* http://usjgofs.whoi.edu/jg/dir/jgofs/southern/nbp97_8/ (2005).

[266] Morrison, J. Final version CTD, including beam attenuation optics. *USJGOFS* http://usjgofs.whoi.edu/jg/dir/jgofs/southern/nbp97_8/ (2005).

[267] Anderson, B. Shipboard underway data. *USJGOFS* http://usjgofs.whoi.edu/jg/dir/jgofs/southern/nbp97_8/ (2005).

[268] Smith, W. O. Fluorometric chlorophyll-a & phaeopigments. *USJGOFS* http://usjgofs.whoi.edu/jg/dir/jgofs/southern/nbp97_8/ (2005).

[269] Bidigare, R. R. Pigments, HPLC method, sampled from bottle and TM casts. *USJGOFS* http://usjgofs.whoi.edu/jg/dir/jgofs/southern/nbp97_8/ (2005).

[270] Smith, W. O. Particulate organic carbon, nitrogen from CTD casts. *USJGOFS* http://usjgofs.whoi.edu/jg/dir/jgofs/southern/nbp97_8/ (2005).

[271] Dunbar, R. NBP9709. *R2R*10.7284/905430 (1997).

[272] Honjo, S. Directory of data in /jgofs/southern/nbp98_2/. *USJGOFS* http://usjgofs.whoi.edu/jg/dir/jgofs/southern/nbp98_2/ (2005).

[273] Program, USA R/V Nathaniel B. Palmer cruise history. *USJGOFS* https://www.usap.gov/usapgov/vesselscienceandoperations/ (2020).

[274] Morrison, J. Final version CTD, including beam attenuation optics. *USJGOFS* http://usjgofs.whoi.edu/jg/dir/jgofs/southern/nbp98_2/ (2005).

[275] Anderson, B. Shipboard underway data. *USJGOFS* http://usjgofs.whoi.edu/jg/dir/jgofs/southern/nbp98_2/ (2005).

[276] Smith, W. O. Fluorometric chlorophyll-a & phaeopigments. *USJGOFS* http://usjgofs.whoi.edu/jg/dir/jgofs/southern/nbp98_2/ (2005).

[277] Smith W, Asper V (2003). NBP0301B field data. R2R.

[278] Smith W (2003). NBP0305A field data. R2R.

[279] Smith, W. Underway hydrographic, weather and ship-state data (JGOFS) from Nathaniel B. Palmer expedition NBP0305A (2003). *MDGS*10.1594/IEDA/315904 (2010).

[280] Arnold G (2005). NBP0501 field data. R2R.

[281] Arnold G (2005). Underway hydrographic, weather and ship-state data (JGOFS) from Nathaniel B. Palmer expedition NBP0501. MDGS.

[282] Smith W (2006). NBP0601A field data. R2R.

[283] Smith W (2008). Uncalibrated hydrographic data acquired with a CTD in the Ross Sea during the Nathaniel B. Palmer expedition NBP0601A. MDGS.

[284] Smith W (2008). Underway hydrographic, weather and ship-state data (JGOFS) from Nathaniel B. Palmer expedition NBP0601A. MDGS.

[285] Gregory MB (2015). Calibrated hydrographic data acquired with a CTD from the Scotia Sea, Antarctica acquired during the Nathaniel B. Palmer expedition NBP0606. MDGS.

[286] Gregory, M. B. Biological dataset collected from bottle casts from the R/V Lawrence M. Gould and the R/V Nathaniel B. Palmer in the Southern Drake Passage and Scotia Sea in support of National Science Foundation projects OPP 03-30443 and ANT 04-44134 from 15 February 2004 to 09 August 2006 (NCEI Accession 0049902). *NCEI* https://accession.nodc.noaa.gov/0049902 (2011).

[287] Gregory, M. B. Measurements taken in the blue water zone (BWZ) under NSF funding near Antarctica and Drakes Passage in 2004 to 2006. *SEABASS* https://10.0.19.203/SeaBASS/NSF-BWZ/DATA001 (2016).

[288] Repository RD (2014). NBP1102. R2R.

[289] Swift, J. CTD data from 320620110219. *CCHDO* https://cchdo.ucsd.edu/cruise/320620110219 (2017).

[290] Swift J (2011). Underway hydrographic, weather and ship-state data (JGOFS) from Nathaniel B. Palmer expedition NBP1102. MDGS.

[291] Repository RD (2014). NBP1403. R2R.

[292] Talley L (2017). CTD data from 320620140320. CCHDO.

[293] Chaves J, Freeman S, Mannino A, Novak M (2015). Underway hydrographic, weather and ship-state data (JGOFS) from Nathaniel B. Palmer expedition NBP1403. SEABASS.

[294] Repository RD (2016). NBP1511. R2R.

[295] Kelly M (2018). Calibrated hydrographic data acquired with a CTD from the Southern Ocean acquired during the Nathaniel B. Palmer expedition NBP1511. MDGS.

[296] Kelly M (2018). Underway hydrographic, weather and ship-state data (JGOFS) from the Southern Ocean acquired during the Nathaniel B. Palmer expedition NBP1511. MDGS.

[297] Tatiana R (2017). NBP1701. R2R.

[298] Tatiana R (2019). Calibrated hydrographic data from the Southern Ocean acquired during R/V Nathaniel B. Palmer expedition NBP1701. MDGS.

[299] Tatiana R (2019). Processed underway hydrographic and weather and ship-state data (JGOFS) from the Southern Ocean acquired during R/V Nathaniel B. Palmer expedition NBP1701. MDGS.

[300] Repository RD (2017). NBP1704. R2R.

[301] Ackley S (2019). Calibrated hydrographic data from the Southern Ocean acquired during R/V Nathaniel B. Palmer expedition NBP1704. MDGS.

[302] Ackley S (2019). Raw underway hydrographic and weather and ship-state data (JGOFS) from the Southern Ocean focus site acquired during R/V Nathaniel B. Palmer expedition NBP1704. MDGS.

[303] Repository RD (2017). NBP1704. R2R.

[304] Mecking S (2018). Uncalibrated hydrographic data from the Pacific Ocean (southern) acquired during R/V Nathaniel B. Palmer expedition NBP1706. MDGS.

[305] Mecking S (2018). Raw underway hydrographic and weather and ship-state data (JGOFS) from the Pacific Ocean (southern) acquired during R/V Nathaniel B. Palmer expedition NBP1706. MDGS.

[306] Repository RD (2018). NBP1802. R2R.

[307] Macdonald A, Briggs E (2020). CTD data from 320620140320. CCHDO.

[308] Macdonald A (2018). Raw underway hydrographic and weather and ship-state data (JGOFS) from the Pacific Ocean acquired during R/V Nathaniel B. Palmer expedition NBP1802. MDGS.

[309] Orsi A, Rosso I (2020). Documentation from cruise 325020190403. CCHDO.

[310] Orsi A, Rosso I (2020). CTD data from cruise 325020190403. CCHDO.

[311] Ross, R. M. LMG99-01 weekly sit rep Jan 2-10. *PAL-LTER* https://pal.lternet.edu/publications/station-cruise-reports (1999).

[312] Ross, R. M. LMGould: SciWeekly 25-31 Jan 99. *PAL-LTER* https://pal.lternet.edu/publications/station-cruise-reports (1999).

[313] Ross, R. M. LMGould: SciWeekly 1-8 Feb 99. *PAL-LTER* https://pal.lternet.edu/publications/station-cruise-reports (1999).

[314] Ross, R. M. Science weekly report LMG00-01. *PAL-LTER* https://pal.lternet.edu/publications/station-cruise-reports (2000).

[315] Ross, R. M. LMG weekly science report 9-16 Jan. *PAL-LTER* https://pal.lternet.edu/publications/station-cruise-reports (2000).

[316] Ross, R. M. LM Gould weekly science report 17-22 Jan. *PAL-LTER* https://pal.lternet.edu/publications/station-cruise-reports (2000).

[317] Ross, R. M. LMG01-01 4 -13 Jan 2001 Palmer LTER cruise. *PAL-LTER* https://pal.lternet.edu/publications/station-cruise-reports (2001).

[318] Ross, R. M. LMG01-01 14 -20 Jan 2001 Palmer LTER cruise. *PAL-LTER* https://pal.lternet.edu/publications/station-cruise-reports (2001).

[319] Ross, R. M. LMG01-01 21-27 January Palmer LTER cruise. *PAL-LTER* https://pal.lternet.edu/publications/station-cruise-reports (2001).

[320] Fraser, B., Vernet, M., Quetin, L. B., Ross, R. M. & Ducklow, H. LMG 0701 situation report 07 – 13 January, 2007. *PAL-LTER* https://pal.lternet.edu/publications/station-cruise-reports (2007).

[321] Fraser, B., Vernet, M., Quetin, L. B., Ross, R. M. & Ducklow, H. LMG 0701 situation report 14 – 21 January, 2007. *PAL-LTER* https://pal.lternet.edu/publications/station-cruise-reports (2007).

[322] Fraser, B., Vernet, M., Quetin, L. B., Ross, R. M. & Ducklow, H. LMG 0701 situation report 21 – 28 January, 2007. *PAL-LTER* https://pal.lternet.edu/publications/station-cruise-reports (2007).

[323] Fraser, W. R., Schofield, O., Steinberg, D., Ducklow, H. & Hollibaugh, J. T. Weekly update 1: LMG cruise 11-01 palmer LTER. *PAL-LTER* https://pal.lternet.edu/publications/station-cruise-reports (2011).

[324] Fraser, W. R., Schofield, O., Steinberg, D., Ducklow, H. & Hollibaugh, J. T. Weekly update 2: LMG cruise 11-01 palmer LTER. *PAL-LTER* https://pal.lternet.edu/publications/station-cruise-reports (2011).

[325] Fraser, W. R., Schofield, O., Steinberg, D., Ducklow, H. & Hollibaugh, J. T. Weekly update 3: LMG cruise 11-01 palmer LTER. *PAL-LTER* https://pal.lternet.edu/publications/station-cruise-reports (2011).

[326] Fraser, W. R., Schofield, O., Steinberg, D., Ducklow, H. & Hollibaugh, J. T. Weekly update 4: LMG cruise 11-01 palmer LTER. *PAL-LTER* https://pal.lternet.edu/publications/station-cruise-reports (2011).

[327] Fraser, W. R. *et al*. LMG 12-01: 29 dec. 2011 – 07 february 2012 LTER cruise 20 weekly science report i. *PAL-LTER* https://pal.lternet.edu/publications/station-cruise-reports (2012).

[328] Fraser, W. R. *et al*. LMG 12-01: 29 dec. 2011 – 07 february 2012 LTER cruise 20 weekly science report 2. *PAL-LTER* https://pal.lternet.edu/publications/station-cruise-reports (2012).

[329] Fraser, W. R. *et al*. LMG 12-01: 29 dec. 2011 – 07 february 2012 LTER cruise 20 weekly science report 3. *PAL-LTER* https://pal.lternet.edu/publications/station-cruise-reports (2012).

[330] Fraser, W. R. *et al*. LMG 12-01: 29 dec. 2011 – 07 february 2012 LTER cruise 20 weekly science report 4. *PAL-LTER* https://pal.lternet.edu/publications/station-cruise-reports (2012).

[331] Ducklow, H. *et al*. LMG 13-01: 30 dec. 2012 – 07 february 2013 LTER cruise 21 weekly science report i. *PAL-LTER* https://pal.lternet.edu/publications/station-cruise-reports (2013).

[332] Ducklow, H. *et al*. LMG 13-01: 30 dec. 2012 – 07 february 2013 LTER cruise 21 weekly science report 2. *PAL-LTER* https://pal.lternet.edu/publications/station-cruise-reports (2013).

[333] Ducklow, H. *et al*. LMG 13-01: 30 dec. 2012 – 07 february 2013 LTER cruise 21 weekly science report 3. *PAL-LTER* https://pal.lternet.edu/publications/station-cruise-reports (2013).

[334] Ducklow, H. *et al*. LMG 13-01: 30 dec. 2012 – 07 february 2013 LTER cruise 21 weekly science report 4. *PAL-LTER* https://pal.lternet.edu/publications/station-cruise-reports (2013).

[335] Schofield, O. *et al*. LMG 14-01: 31 dec. 2013 – 05 february 2013 LTER cruise 22 weekly science report 1. *PAL-LTER* https://pal.lternet.edu/publications/station-cruise-reports (2014).

[336] Schofield, O. *et al*. LMG 14-01: 31 dec. 2013 – 05 february 2013 LTER cruise 22 weekly science report 2. *PAL-LTER* https://pal.lternet.edu/publications/station-cruise-reports (2014).

[337] Schofield, O. *et al*. LMG 14-01: 31 dec. 2013 – 05 february 2013 LTER cruise 22 weekly science report 3. *PAL-LTER* https://pal.lternet.edu/publications/station-cruise-reports (2014).

[338] Schofield, O. *et al*. LMG 14-01: 31 dec. 2013 – 05 february 2013 LTER cruise 22 weekly science report 4. *PAL-LTER* https://pal.lternet.edu/publications/station-cruise-reports (2014).

[339] Steinberg, D. *et al*. LMG 15-01: 29 dec. 2014 – 09 february 2015, PAL LTER cruise 23 weekly science report 1. *PAL-LTER* https://pal.lternet.edu/publications/station-cruise-reports (2015).

[340] Steinberg, D. *et al*. LMG 15-01: 29 dec. 2014 – 09 february 2015, PAL LTER cruise 23 weekly science report 2. *PAL-LTER* https://pal.lternet.edu/publications/station-cruise-reports (2015).

[341] Steinberg, D. *et al*. LMG 15-01: 29 dec. 2014 – 09 february 2015, PAL LTER cruise 23 weekly science report 3. *PAL-LTER* https://pal.lternet.edu/publications/station-cruise-reports (2015).

[342] Steinberg, D. *et al*. LMG 15-01: 29 dec. 2014 – 09 february 2015, PAL LTER cruise 23 weekly science report 4. *PAL-LTER* https://pal.lternet.edu/publications/station-cruise-reports (2015).

[343] Fraser, W. R. *et al*. LMG 16-01: 03 jan – 17 feb. 2016 LTER cruise 24 weekly science report 1. *PAL-LTER* https://pal.lternet.edu/publications/station-cruise-reports (2016).

[344] Fraser, W. R. *et al*. LMG 16-01: 03 jan – 17 feb. 2016 LTER cruise 24 weekly science report 2. *PAL-LTER* https://pal.lternet.edu/publications/station-cruise-reports (2016).

[345] Fraser, W. R. *et al*. LMG 16-01: 03 jan – 17 feb. 2016 LTER cruise 24 weekly science report 3. *PAL-LTER* https://pal.lternet.edu/publications/station-cruise-reports (2016).

[346] Fraser, W. R. *et al*. LMG 16-01: 03 jan – 17 feb. 2016 LTER cruise 24 final weekly science report. *PAL-LTER* https://pal.lternet.edu/publications/station-cruise-reports (2016).

[347] Schofield, O., Ducklow, H., Steinberg, D., Fraser, W. R. & Friedlaender, A. Science report for Week 1 of LTER 1701. *PAL-LTER* https://pal.lternet.edu/publications/station-cruise-reports (2017).

[348] Martinson, D. *et al*. Science report for week 2 of LTER 1701. *PAL-LTER* https://pal.lternet.edu/publications/station-cruise-reports (2017).

[349] Martinson, D. *et al*. Science report for week 3 of LTER 1701. *PAL-LTER* https://pal.lternet.edu/publications/station-cruise-reports (2017).

[350] Ducklow H (2019). Raw underway hydrographic and weather and ship-state data (JGOFS) from the Antarctic Peninsula acquired during R/V Laurence M. Gould expedition LMG1701 (2016). MDGS.

[351] Bullister, J. L. & Johnson, G. C. Documentation from cruise 33RO20071215. *CCHDO* https://cchdo.ucsd.edu/cruise/33RO20071215 (2016).

[352] Bullister, J. L. & Johnson, G. C. Ctd data from cruise 33RO20071215. *CCHDO* https://cchdo.ucsd.edu/cruise/33RO20071215 (2016).

[353] Wanninkhof R (2018). Underway physical oceanography and carbon dioxide measurements during Ronald H. Brown cruise 33RO20071215. CCHDO.

[354] Feely RA (2013). Partial pressure (or fugacity) of carbon dioxide, dissolved inorganic carbon, pH, alkalinity, temperature, salinity and other variables collected from discrete sample and profile observations using alkalinity titrator, CTD and other instruments from NOAA ship Ronald H. Brown in the north Pacific Ocean, south Pacific Ocean and southern oceans from 2007-12-15 to 2008-02-23 (NCEI accession 0109903). NCEI.

[355] Sonnerup, R., Carter, B., Purkey, S. & Bourbonnais, A. Documentation from cruise 33RO20161119. *CCHDO* https://cchdo.ucsd.edu/cruise/33RO20161119 (2020).

[356] Sonnerup, R., Carter, B., Purkey, S. & Bourbonnais, A. CTD data from cruise 33RO20161119. *CCHDO* https://cchdo.ucsd.edu/cruise/33RO20161119 (2020).

[357] Morison, J. Final CTD data, including beam attenuation optics. *USJGOFS* http://usjgofs.whoi.edu/jg/dir/jgofs/southern/rr-kiwi_6/ (2005).

[358] Goericke, R. Pigments, HPLC method, sampled from bottle casts. *USJGOFS* http://usjgofs.whoi.edu/jg/dir/jgofs/southern/rr-kiwi_6/ (2005).

[359] Smith, W. O. Fluorometric chlorophyll-a. *USJGOFS* http://usjgofs.whoi.edu/jg/dir/jgofs/southern/rr-kiwi_6/ (2005).

[360] Smith, W. O. Particulate organic carbon, nitrogen from CTD casts. *USJGOFS* http://usjgofs.whoi.edu/jg/dir/jgofs/southern/rr-kiwi_6/ (2005).

[361] Morison, J. Final CTD data, including beam attenuation optics. *USJGOFS* http://usjgofs.whoi.edu/jg/dir/jgofs/southern/rr-kiwi_7/ (2005).

[362] Goericke, R. Pigments, HPLC method, sampled from bottle casts. *USJGOFS* http://usjgofs.whoi.edu/jg/dir/jgofs/southern/rr-kiwi_7 (2005).

[363] Smith, W. O. Fluorometric chlorophyll-a. *USJGOFS* http://usjgofs.whoi.edu/jg/dir/jgofs/southern/rr-kiwi_7/ (2005).

[364] Smith, W. O. Particulate organic carbon, nitrogen from CTD casts. *USJGOFS* http://usjgofs.whoi.edu/jg/dir/jgofs/southern/rr-kiwi_7/ (2005).

[365] Morison, J. Final CTD data, including beam attenuation optics. *USJGOFS* http://usjgofs.whoi.edu/jg/dir/jgofs/southern/rr-kiwi_8/ (2005).

[366] Morison, J. Processed CTD data from TM casts. *USJGOFS* http://usjgofs.whoi.edu/jg/dir/jgofs/southern/rr-kiwi_8/ (2005).

[367] Goericke, R. Pigments, HPLC method, sampled from bottle casts. *USJGOFS* http://usjgofs.whoi.edu/jg/dir/jgofs/southern/rr-kiwi_8/ (2005).

[368] Smith, W. O. Fluorometric chlorophyll-a. *USJGOFS* http://usjgofs.whoi.edu/jg/dir/jgofs/southern/rr-kiwi_8/ (2005).

[369] Smith, W. O. Particulate organic carbon, nitrogen from CTD casts. *USJGOFS* http://usjgofs.whoi.edu/jg/dir/jgofs/southern/rr-kiwi_8/ (2005).

[370] Morison, J. Final CTD data, including beam attenuation optics. *USJGOFS* http://usjgofs.whoi.edu/jg/dir/jgofs/southern/rr-kiwi_9/ (2005).

[371] Goericke, R. Pigments, HPLC method, sampled from bottle casts. *USJGOFS* http://usjgofs.whoi.edu/jg/dir/jgofs/southern/rr-kiwi_9/ (2005).

[372] Smith, W. O. Fluorometric chlorophyll-a. *USJGOFS* http://usjgofs.whoi.edu/jg/dir/jgofs/southern/rr-kiwi_9/ (2005).

[373] Smith, W. O. Particulate organic carbon, nitrogen from CTD casts. *USJGOFS* http://usjgofs.whoi.edu/jg/dir/jgofs/southern/rr-kiwi_9/ (2005).

[374] Buesseler, K. O., K. H. C. & Siegel, D. USCGC Polar Star cruises COOK19MV, DRFT08RR, PS02_2002 from the Southern Ocean, south of New Zealand in 2002 (SOFeX project). *BCO-DMO* http://lod.bco-dmo.org/id/dataset/2851 (2007).

[375] Bidigare, R. R. & Landry, M. R. Pigments from HPLC analysis of bottle samples from SOFeX cruisse PS02_2002, COOK19MV, DRFT08RR from the Southern Ocean, south of New Zealand, Southern Ocean in 2002 (SOFeX project). *BCO-DMO* http://lod.bco-dmo.org/id/dataset/2851 (2007).

[376] Richard Barber, F. C. & Coale, K. H. Fluorometric chlorophyll from Niskin and TM bottle samples from R/V Melville, R/V Roger Revelle cruises COOK19MV, DRFT08RR from the Southern Ocean, south of New Zealand in 2002 (SOFeX project). *BCO-DMO* http://lod.bco-dmo.org/id/dataset/2933 (2008).

[377] Altabet, M. A. & Timothy, D. Particulate organic matter (POM) from R/V Melville, R/V Roger Revelle cruises COOK19MV, DRFT08RR from the Southern Ocean, south of New Zealand in 2002 (SOFeX project). *BCO-DMO* http://lod.bco-dmo.org/id/dataset/2926 (2007).

[378] Sloyan, B. M. & Swift, J. Documentation from cruise 33RR200501. *CCHDO* https://cchdo.ucsd.edu/cruise/33RR200501 (2015).

[379] Sloyan, B. M. & Swift, J. Ctd data from cruise 33RR200501. *CCHDO* https://cchdo.ucsd.edu/cruise/33RR200501 (2017).

[380] Swift, J. Documentation from cruise 33RR20070204. *CCHDO* https://cchdo.ucsd.edu/cruise/33RR20070204 (2017).

[381] Swift, J. CTD data from cruise 33RR20070204. *CCHDO* https://cchdo.ucsd.edu/cruise/33RR20070204 (2017).

[382] Speer, K. G. & Dittmar, T. Documentation from cruise 33RR20080204. *CCHDO* https://cchdo.ucsd.edu/cruise/33RR20080204 (2009).

[383] Speer, K. G. Ctd data from cruise 33RR20080204. *CCHDO* https://cchdo.ucsd.edu/cruise/33RR20080204 (2017).

[384] Wanninkhof R (2016). PANGAEA.

[385] Macdonald A (2016). Documentation from cruise 33RR20160208. CCHDO.

[386] Macdonald A (2016). Ctd data from cruise 33RR20160208. CCHDO.

[387] Wanninkhof R, Pierrot D (2017). PANGAEA.

[388] Macdonald, A. M. *et al*. Discrete profile measurements of dissolved inorganic carbon, total alkalinity, pH and other hydrographic and chemical data obtained during the R/V Roger Revelle repeat hydrography and SOCCOM cruise in the Indian Ocean and Southern Ocean: GO-SHIP section I08S (EXPOCODE 33RR20160208), from 2016-02-08 to 2016-03-16 (NCEI accession 0171457). *NCEI*10.7289/v5hm56qr (2018).

[389] Wright S (2013). Australian Antarctic Data Centre.

[390] Wright S (2013). Australian Antarctic Data Centre.

[391] McMinn, A. Fast repetition rate fluorometry (FRRF) data collected on the RV l’astrolabe. *IMAS* https://metadata.imas.utas.edu.au/geonetwork/srv/eng/catalog.search#/metadata/fc64fc7b-29b0-4607-9527-899dfa991d68 (2011).

[392] Wright S (2013). Australian Antarctic Data Centre.

[393] Wright S (2013). Australian Antarctic Data Centre.

[394] Wright S (2013). Australian Antarctic Data Centre.

[395] Wright S (2013). Australian Antarctic Data Centre.

[396] Wright S (2013). Australian Antarctic Data Centre.

[397] Wright S (2013). Australian Antarctic Data Centre.

[398] Wright S (2013). Australian Antarctic Data Centre.

[399] Wright S (2013). Australian Antarctic Data Centre.

[400] Wright S (2013). Australian Antarctic Data Centre.

[401] Wright S (2013). Australian Antarctic Data Centre.

[402] Wright S (2013). Australian Antarctic Data Centre.

[403] Wright S (2013). Australian Antarctic Data Centre.

[404] Wright S (2013). Australian Antarctic Data Centre.

[405] Wright S (2013). Australian Antarctic Data Centre.

[406] Wright S (2013). Australian Antarctic Data Centre.

[407] Wright S (2013). Australian Antarctic Data Centre.

[408] Wright S (2013). Australian Antarctic Data Centre.

[409] Wright S (2013). Australian Antarctic Data Centre.

[410] Wright S (2013). Australian Antarctic Data Centre.

[411] Fiala M (1994). ANTARES 2-MD78/ANTARFIX 4 cruise, RV Marion Dufresne. SISMER.

[412] Fiala M, French CTD (1994). data from ANTARES 2-MD78/ANTARFIX 4 cruise. SISMER.

[413] Mayzaud P (1995). ANTARES 3 cruise, RV Marion Dufresne. SISMER.

[414] Mayzaud P, French CTD (1995). data from ANTARES 3 cruise. SISMER.

[415] Marty J-C (2004). PANGAEA.

[416] Marty J-C (2004). PANGAEA.

[417] Marty J-C (2004). PANGAEA.

[418] Marty J-C (2004). PANGAEA.

[419] Marty J-C (2004). PANGAEA.

[420] Marty J-C (2004). PANGAEA.

[421] Descolas-Gros C, Edwige C (2012). PANGAEA.

[422] Claire Lo Monaco, N. M. Dissolved inorganic carbon (DIC), total alkalinity, temperature, salinity and other variables collected from discrete sample and profile observations during the R/V Marion Dufresne cruise OISO-01 (EXPOCODE 35MF19980121) in the Indian Ocean from 1998-01-21 to 1998-02-19 (NCEI accession 0113570). *NCEI*10.3334/CDIAC/otg.CARINA_35MF19980121 (2013).

[423] Claire Lo Monaco, N. M. Dissolved inorganic carbon (DIC), total alkalinity, temperature, salinity and other variables collected from discrete sample and profile observations during the R/V Marion Dufresne cruise OISO-02 (EXPOCODE 35MF19980818) in the Indian Ocean from 1998-08-18 to 1998-09-09 (NCEI accession 0113573). *NCEI*10.3334/CDIAC/otg.CARINA_35MF19980818 (2013).

[424] Claire Lo Monaco, N. M. Dissolved inorganic carbon (DIC), total alkalinity, temperature, salinity and other variables collected from discrete sample and profile observations during the R/V Marion Dufresne cruise OISO-03 (EXPOCODE 35MF19981205) in the Indian Ocean from 1998-12-05 to 1998-12-27 (NCEI accession 0113574). *NCEI*10.3334/CDIAC/otg.CARINA_35MF19981205 (2013).

[425] Claire Lo Monaco, N. M. Dissolved inorganic carbon (DIC), total alkalinity, temperature, salinity and other variables collected from discrete sample and profile observations during the R/V Marion Dufresne cruise OISO-04 (EXPOCODE 35MF20000117) in the Indian Ocean from 2000-01-17 to 2000-01-27 (NCEI accession 0113575). *NCEI*10.3334/CDIAC/otg.CARINA_35MF20000117 (2013).

[426] Claire Lo Monaco, N. M. Dissolved inorganic carbon (DIC), total alkalinity, temperature, salinity and other variables collected from discrete sample and profile observations during the R/V Marion Dufresne cruise OISO-05 (EXPOCODE 35MF20010103) in the Indian Ocean from 2000-07-19 to 2000-08-16 (NCEI accession 0113576). *NCEI*10.3334/CDIAC/otg.CARINA_35MF20000719 (2013).

[427] Claire Lo Monaco, N. M. Dissolved inorganic carbon (DIC), total alkalinity, temperature, salinity and other variables collected from discrete sample and profile observations during the R/V Marion Dufresne cruise OISO-06 (EXPOCODE 35MF20010103) in the Indian Ocean from 2001-01-03 to 2001-01-26 (NCEI accession 0113577). *NCEI*10.3334/CDIAC/otg.CARINA_35MF20010103 (2013).

[428] Claire Lo Monaco, N. M. Dissolved inorganic carbon (DIC), total alkalinity, temperature, salinity and other variables collected from discrete sample and profile observations during the R/V Marion Dufresne cruise OISO-08 (EXPOCODE 35MF20020104) in the Indian Ocean from 2002-01-04 to 2002-02-01 (NCEI accession 0113578). *NCEI*10.3334/CDIAC/otg.CARINA_35MF20020104 (2013).

[429] Metzl N (2004). VT 62/CARAUS - OISO 11 cruise, RV Marion Dufresne. SISMER.

[430] Metzl N (2004). French CTD data from VT 62/CARAUS - OISO 11 cruise. SISMER.

[431] Claire Lo Monaco, N. M. Dissolved inorganic carbon (DIC), total alkalinity, temperature, salinity and other variables collected from discrete sample and profile observations during the R/V Marion Dufresne cruise OISO-11 (EXPOCODE 35MF20040103) in the indian ocean from 2004-01-03 to 2004-02-09 (NCEI accession 0113572). *NCEI*10.3334/cdiac/otg.carina_35mf20040103 (2013).

[432] Peeken I, Bluhm K, Zöllner E (2017). PANGAEA.

[433] Picheral M (2014). PANGAEA.

[434] Claustre H (2013). PANGAEA.

[435] Masao Ishii, M. T. Dissolved inorganic carbon, temperature, salinity and other variables collected from discrete sample and profile observations using CTD, bottle and other instruments from the Hakuho Maru in the Indian Ocean, north Pacific Ocean and south pacific ocean from 2001-12-08 to 2002-01-19 (NCEI accession 0112347). *NCEI*10.3334/CDIAC/otg.CARINA_49HH20011127 (2013).

[436] Fukasawa, M. Documentation from cruise 49NZ20030803. *CCHDO* https://cchdo.ucsd.edu/cruise/49NZ20030803 (2015).

[437] Fukasawa, M. CTD data from cruise 49NZ20030803. *CCHDO* https://cchdo.ucsd.edu/cruise/49NZ20030803 (2015).

[438] Clemenston, L. Data collected on leg 1 of the BEAGLE cruises during August/September 2003. *CCHDO* https://catalogue-imos.aodn.org.au/geonetwork/srv/en/metadata.show?uuid=97b9fe73-ee44-437f-b2ae-5b8613f81042 (2011).

[439] Watanabe, S. Documentation from cruise 49NZ20030909. *CCHDO* https://cchdo.ucsd.edu/cruise/49NZ20030909 (2015).

[440] Watanabe, S. CTD data from cruise 49NZ20030909. *CCHDO* https://cchdo.ucsd.edu/cruise/49NZ20030909 (2015).

[441] Clemenston, L. Data collected on leg 2 of the BEAGLE cruises during September/October 2003. *CCHDO* https://catalogue-imos.aodn.org.au/geonetwork/srv/en/metadata.show?uuid=97b9fe73-ee44-437f-b2ae-5b8613f81042 (2011).

[442] Clemenston, L. Data collected on leg 3 of the BEAGLE cruises during October 2003. *CCHDO* https://catalogue-imos.aodn.org.au/geonetwork/srv/en/metadata.show?uuid=97b9fe73-ee44-437f-b2ae-5b8613f81042 (2011).

[443] Yoshikawa, Y. Documentation from cruise 49NZ20031106. *CCHDO* https://cchdo.ucsd.edu/cruise/49NZ20031106 (2015).

[444] Yoshikawa, Y. CTD data from cruise 49NZ20031106. *CCHDO* https://cchdo.ucsd.edu/cruise/49NZ20031106 (2015).

[445] Clemenston, L. Data collected on leg 4 of the BEAGLE cruises during November/December 2003. *CCHDO* https://catalogue-imos.aodn.org.au/geonetwork/srv/en/metadata.show?uuid=97b9fe73-ee44-437f-b2ae-5b8613f81042 (2011).

[446] Fukasawa, M. Documentation from cruise 49NZ20031209. *CCHDO* https://cchdo.ucsd.edu/cruise/49NZ20031209 (2015).

[447] Fukasawa, M. CTD data from cruise 49NZ20031209. *CCHDO* https://cchdo.ucsd.edu/cruise/49NZ20031209 (2015).

[448] Clemenston, L. Data collected on leg 5 of the BEAGLE cruises between December 2003 and January 2004. *CCHDO* https://catalogue-imos.aodn.org.au/geonetwork/srv/en/metadata.show?uuid=97b9fe73-ee44-437f-b2ae-5b8613f81042 (2011).

[449] Clemenston, L. Data collected on leg 6 of the BEAGLE cruises during January and February 2004. *CCHDO* https://catalogue-imos.aodn.org.au/geonetwork/srv/en/metadata.show?uuid=97b9fe73-ee44-437f-b2ae-5b8613f81042 (2011).

[450] Katsumata, K. Documentation from cruise 49NZ20121128. *CCHDO* https://cchdo.ucsd.edu/cruise/49NZ20121128 (2015).

[451] Katsumata, K. CTD data from cruise 49NZ20121128. *CCHDO* https://cchdo.ucsd.edu/cruise/49NZ20121128 (2015).

[452] Katsumata, K. *et al*. Dissolved inorganic carbon (DIC), pH on total scale, total alkalinity, temperature, salinity and other variables collected from discrete samples and profile observations during the R/V Mirai cruise GO-SHIP_P14S_S04_2012; MR12-05 leg 2 (EXPOCODE 49NZ20121128) in the Indian, south Pacific and Southern Oceans from 2012-11-28 to 2013-01-04 (NCEI accession 0143950). *NCEI*10.3334/cdiac/otg.goship_p14s_s04_2012 (2016).

[453] Uchida, H. Documentation from cruise 49NZ20130106. *CCHDO* https://cchdo.ucsd.edu/cruise/49NZ20130106 (2015).

[454] Uchida, H. CTD data from cruise 49NZ20130106. *CCHDO* https://cchdo.ucsd.edu/cruise/49NZ20130106 (2015).

[455] Uchida, H., Murata, A., Aoyama, M., Kumamoto, Y. & Aoki, S. Dissolved inorganic carbon (DIC), pH on total scale, total alkalinity, temperature, salinity and other variables collected from discrete sample and profile observations obtained during the R/V Mirai cruise GOSHIP_S04I_2013 (EXPOCODE 49NZ20130106) in the Indian Ocean and southern oceans from 2013-01-06 to 2013-02-15 (NCEI accession 0156925). *NCEI*10.3334/CDIAC/OTG.GOSHIP_S04I_2013 (2016).

[456] Uchida, H. Documentation from cruise 49NZ20170208. *CCHDO* https://cchdo.ucsd.edu/cruise/49NZ2017020 (2018).

[457] Uchida, H. CTD data from cruise 49NZ20170208. *CCHDO* https://cchdo.ucsd.edu/cruise/49NZ20170208 (2018).

[458] Uchida, H., Murata, A., Kumamoto, Y., Aoyama, M. & Sasaki, K. Dissolved inorganic carbon (DIC), total alkalinity, chlorofluorocarbons (CFC-11, CFC-12, CFC113), temperature, salinity, oxygen, nutrients and other measurements collected from profile and discrete sample observations during the R/V Mirai cruise MR16-09, GO-SHIP_P17E (EXPOCODE 49NZ20170208) in the south pacific ocean from 2017-02-08 to 2017-03-05 (NCEI accession 0186207). *NCEI*10.25921/zmae-wa37 (2019).

[459] Masao Ishii, R. M., Naoki Sato. Dissolved inorganic carbon, pH, temperature, salinity and other variables collected from discrete sample and profile observations using CTD, bottle and other instruments from the SHIRASE in the Indian Ocean, south Pacific Ocean and Tasman Sea from 1992-12-03 to 1993-03-19 (NCEI accession 0113597). *NCEI*10.3334/CDIAC/otg.CARINA_49ZS19921203 (2013).

[460] Tsuneo Odate, M. Ishii; Dissolved inorganic carbon, temperature, salinity and other variables collected from discrete sample and profile observations using CTD, bottle and other instruments from the TANGAROA in the Indian Ocean from 2003-02-17 to 2003-03-12 (NCEI accession 0113599). *NCEI*10.3334/cdiac/otg.carina_61tg20030217 (2013).

[461] Owens, N. J. P. Documentation from cruise 74JC002_1. *CCHDO* https://cchdo.ucsd.edu/cruise/74JC002_1 (2015).

[462] Owens, N. J. P. CTD data from cruise 74JC002_1. *CCHDO* https://cchdo.ucsd.edu/cruise/74JC002_1 (2015).

[463] Firing, Y. Documentation from cruise 74JC20151217. *CCHDO* https://cchdo.ucsd.edu/cruise/74JC20151217 (2016).

[464] Firing, Y. CTD data from cruise 74JC20151217. *CCHDO* https://cchdo.ucsd.edu/cruise/74JC20151217 (2017).

[465] de Villiers S, Siswana K, Vena K (2015). PANGAEA.

[466] Olsen A (2016). The global ocean data analysis project version 2 (GLODAPv2) – an internally consistent data product for the world ocean. Earth Syst. Sci. Data.

[467] Key RM (2015). Global ocean data analysis project, version 2 (GLODAPv2). NCEI.

[468] System, I. M. O. IMOS - SRS - ocean colour - bio optical database of australian waters. *AODN* https://catalogue-imos.aodn.org.au/geonetwork/srv/en/metadata.show?uuid=97b9fe73-ee44-437f-b2ae-5b8613f81042 (2019).

[469] Peloquin JM (2013). PANGAEA.

[470] Peloquin JM (2013). The MAREDAT global database of high performance liquid chromatography marine pigment measurements. Earth Syst. Sci. Data.

[471] Taylor BB (2011). Bio-optical provinces in the eastern Atlantic Ocean and their biogeographical relevance. Biogeosci..

[472] Hoffmann LJ, Peeken I, Lochte K, Assmy P, Veldhuis M (2006). Different reactions of Southern Ocean phytoplankton size classes to iron fertilization. Limnol. Oceanogr..

[473] Yentsch CS, Menzel DW (1963). A method for the determination of phytoplankton chlorophyll and phaeophytin by fluorescence. Deep Sea Res. and Oceanog. Abstracts.

[474] Ras J, Claustre H, Uitz J (2008). Spatial variability of phytoplankton pigment distributions in the subtropical south Pacific Ocean: Comparison between in situ and predicted data. Biogeosci..

[475] Welschmeyer NA (1994). Fluorometric analysis of chlorophyll a in the presence of chlorophyll b and pheopigments. Limnol. and Oceanogr..

[476] Neveux J, Panouse M (1987). Spectrofluorometric determination of chlorophylls and pheophytin. Archiv fiir Hydrobiologie.

[477] Lorenzen CJ (1967). Determination of chlorophyll and pheo-pigments: spectrophotometric equations. Limnol. and Oceanogr..

[478] Bidigare, R. R., Van Heukelem, L. & Trees, C. C. Analysis of algal pigments by high-performance liquid chromatography. *Algal culturing techniques* 327–345 (2005).

[479] Mueller, J. L. *et al*. Ocean optics protocols for satellite ocean color sensor validation, revision 4. Volume III: Radiometric measurements and data analysis protocols. *Goddard Space Flight Space Centre* 1–63 10.25607/OBP-62 (2003).

[480] Van Heukelem L, Thomas CS (2001). Computer-assisted high-performance liquid chromatography method development with applications to the isolation and analysis of phytoplankton pigments. J. Chromatogr. A.

[481] Hooker, S. *et al*. The fifth SeaWiFS HPLC analysis round-robin experiment (SeaHARRE-5). *NASA* 1–108 (2012).

[482] Álvarez E, Thoms S, Bracher A, Liu Y, Völker C (2019). Modeling photoprotection at global scale: The relative role of nonphotosynthetic pigments, physiological state, and species composition. Global Biogeochem. Cycles.

[483] Wright SW (2010). Phytoplankton community structure and stocks in the Southern Ocean (30–80°e) determined by CHEMTAX analysis of HPLC pigment signatures. Deep Sea Res. 2 Top. Stud. Oceanogr..

[484] Smith WO (2006). Interannual variations in nutrients, net community production, and biogeochemical cycles in the ross sea. Deep Sea Res. 2 Top. Stud. Oceanogr..

[485] Wright SW (1991). Improved HPLC method for the analysis of chlorophylls and carotenoids from marine phytoplankton. Mar. Ecol. Prog. Ser..

[486] Peloquin J (2013). The MAREDAT global database of high performance liquid chromatography marine pigment measurements. Earth Syst. Sci. Data.

[487] Villiers S, de, Siswana K, Vena K (2015). In situ measurement of the biogeochemical properties of southern ocean mesoscale eddies in the southwest Indian Ocean, April 2014. Earth Syst. Sci. Data.

[488] Holm-Hansen O, Riemann B (1978). Chlorophyll a determination: Improvements in methodology. Oikos.

[489] Ishii Masao, M. F., T Suzuki. Report on the surface phytoplankton pigments measured during the JARE-34 cruise to syowa station, antarctica, november 1992 to march 1993. *JARE data reports*. *Marine biology*10.15094/00003303 (1996).

[490] Jeffrey SW, Humphrey GF (1975). New spectrophotometric equations for determining chlorophylls a, b, c1 and c2 in higher plants, algae and natural phytoplankton. Biochemie und Physiologie der Pflanzen.

[491] Intergovernmental Oceanographic Commission (1994). Protocols for the joint global ocean flux study (JGOFS) core measurements. Intergovernmental Oceanographic Commission Manuals and Guides.

[492] Smetacek, V., Bathmann, U. & El Naggar, S. E. D. The expeditions ANTARKTIS XVIII/1-2 of the research vessel. *Reports on Polar and Marine Research* **400** (2001).

[493] Carbotte SM (2007). Antarctic multibeam bathymetry and geophysical data synthesis: An On-Line Digital Data Resource for Marine Geoscience Research in the Southern Ocean. USGS.

[494] US Antarctic Program R/V Laurence M. Gould cruise history. https://www.usap.gov/usapgov/vesselscienceandoperations/ (2020).

[495] Iannuzzi, R. Conductivity temperature depth (CTD) sensor profile data binned by depth from PAL LTER annual cruises, 1991 - 2017 (ongoing). *PAL-LTER*10.6073/pasta/12276fbc0d68568177702aed0d4b44bc (2018).

[496] Oscar Schofield, B. P., Vernet, M. Photosynthetic pigments of water column samples and analyzed with high performance liquid chromatography (HPLC), collected aboard Palmer LTER annual cruises off the coast of the Western Antarctica Peninsula, 1991 - 2016. *PAL-LTER*10.6073/pasta/4d583713667a0f52b9d2937a26d0d82e (2018).

[497] Oscar Schofield, R. S., Vernet, M. Chlorophyll and phaeopigments from water column samples, collected at selected depths aboard Palmer LTER annual cruises off the coast of the Western Antarctic Peninsula, 1991 – 2019. *PAL-LTER*10.6073/pasta/6bbce1e3264571463c0354874dba88cf (2020).

[498] Hugh Ducklow BP, Vernet M (2017). Particulate organic carbon and nitrogen measurements from water column sample bottles, collected aboard Palmer LTER annual cruises off the Western Antarctic Peninsula, 1991 - 2018. Cruise PD94-01 not included in time series for lack of samples. PAL-LTER.

[499] Walton, D. & Klepikov, A. CTD data from cruise RUB320161220. *CCHDO* https://cchdo.ucsd.edu/cruise/RUB320161220 (2019).

[500] Werdell PJ (2003). Unique data repository facilitates ocean color satellite validation. Eos, Transactions American Geophysical Union.

[501] Werdell, P. J. & Bailey, S. The SeaWiFS bio-optical archive and storage system (SeaBASS): Current architecture and implementation. *NASA* 1–45 (2002).

[502] Boss E, Talley L (2020). CLIVAR. SeaBASS.

[503] Boss E, Talley L (2020). SOCCOM HPLC pigments. SeaBASS.

[504] Arrigo K (2020). Measurements taken off the Antarctic coast in the ross sea between 1996 and 1998 under the research on ocean-atmosphere variability and ecosystem response in the ross sea (ROAVERRS). SeaBASS.

[505] Smith W (2017). 0. Bottle data including phosphate, nitrate, total nitrite and nitrate, ammonium, silicate, chlorophyll, particulate organic carbon and nitrogen (POC, PON), and biogenic silica from multiple cruises to the southern Ross Sea, 2001-2006 (IVARS project). BCO-DMO.

[506] Felden J (2023). PANGAEA – Data Publisher for Earth & Environmental Science. Sci. Data.

[507] Haëntjens N, Boss E, Talley LD (2017). Revisiting ocean color algorithms for chlorophyll a and particulate organic carbon in the southern ocean using biogeochemical floats. J. Geophys. Res. Oceans.

[508] Bianchi TS, Lambert C, Biggs DC (1995). Distribution of chlorophyll a and phaeopigments in the northwestern Gulf of Mexico: A comparison between fluorometric and high-performance liquid chromatography measurements. Bull. Mar. Sci..

[509] Dos Santos ACA (2003). Comparison of three methods for chlorophyll determination: Spectrophotometry and fluorimetry in samples containing pigment mixtures and spectrophotometry in samples with separate pigments through high performance liquid chromatography. Acta Limnol. Bras.

[510] Kumari B (2005). Comparison of high performance liquid chromatography and fluorometric ocean colour pigments. J. Indian Soc. Rem. Sens..

[511] Kratzer S, Harvey ET, Canuti E (2022). International intercomparison of in situ chlorophyll-a measurements for data quality assurance of the Swedish Monitoring Program. Front. Rem. Sens..

